# Copy-number variants in clinical genome sequencing: deployment and interpretation for rare and undiagnosed disease

**DOI:** 10.1038/s41436-018-0295-y

**Published:** 2018-10-08

**Authors:** Andrew M. Gross, Subramanian S. Ajay, Vani Rajan, Carolyn Brown, Krista Bluske, Nicole J. Burns, Aditi Chawla, Alison J. Coffey, Alka Malhotra, Alicia Scocchia, Erin Thorpe, Natasa Dzidic, Karine Hovanes, Trilochan Sahoo, Egor Dolzhenko, Bryan Lajoie, Amirah Khouzam, Shimul Chowdhury, John Belmont, Eric Roller, Sergii Ivakhno, Stephen Tanner, Julia McEachern, Tina Hambuch, Michael Eberle, R. Tanner Hagelstrom, David R. Bentley, Denise L. Perry, Ryan J. Taft

**Affiliations:** 1 Illumina Inc., San Diego, CA USA; 2 CombiMatrix Diagnostics (currently Invitae), Irvine, CA USA; 3 Invitae Corporation, San Francisco, CA USA; 4 Rady Children’s Institute for Genomic Medicine and Rady Children’s Hospital, Encinitas CA, USA; 5 Illumina Cambridge Ltd., Little Chesterford, UK

**Keywords:** whole genome sequencing (WGS), copy number variation (CNV), rare and undiagnosed disease, structural variation (SV), microarray

## Abstract

**Purpose:**

Current diagnostic testing for genetic disorders involves serial use of specialized assays spanning multiple technologies. In principle, genome sequencing (GS) can detect all genomic pathogenic variant types on a single platform. Here we evaluate copy-number variant (CNV) calling as part of a clinically accredited GS test.

**Methods:**

We performed analytical validation of CNV calling on 17 reference samples, compared the sensitivity of GS-based variants with those from a clinical microarray, and set a bound on precision using orthogonal technologies. We developed a protocol for family-based analysis of GS-based CNV calls, and deployed this across a clinical cohort of 79 rare and undiagnosed cases.

**Results:**

We found that CNV calls from GS are at least as sensitive as those from microarrays, while only creating a modest increase in the number of variants interpreted (~10 CNVs per case). We identified clinically significant CNVs in 15% of the first 79 cases analyzed, all of which were confirmed by an orthogonal approach. The pipeline also enabled discovery of a uniparental disomy (UPD) and a 50% mosaic trisomy 14. Directed analysis of select CNVs enabled breakpoint level resolution of genomic rearrangements and phasing of de novo CNVs.

**Conclusion:**

Robust identification of CNVs by GS is possible within a clinical testing environment.

## Introduction

Variation in DNA copy number is a well-described cause of human genetic disease.^1^ Copy-number variants (CNVs) associated with human pathologies range from chromosomal aneuploidy, to microduplication and microdeletion syndromes, and include smaller structural variants (SVs) that affect single genes and exons.^1–5^ Karyotype and microarray analyses have served as gold standards in molecular diagnostics for CNVs, but the increasing number and complexity of possible genomic changes requires testing that can simultaneously address the complete range of cytogenetic abnormalities and smaller SVs.

Genome sequencing (GS) can be used to detect almost all classes of alleles. It is sensitive and specific for single-nucleotide variants (SNVs) and indels^6–8^, and proof-of-principle studies have shown the ability to detect complex repeat expansions,^9^ CNVs,^10,11^ and SVs.^12^ Approaches have been developed to enable CNV detection as a component of gene panel or exome sequencing analyses, which have improved diagnostic yield.^13–15^ Despite this success, such targeted approaches have technical limitations arising from nonuniform sequencing depth, polymerase chain reaction (PCR) artifacts, GC bias, and a high variance in allele fraction.^16–18^ In contrast, GS sequencing depth is predictable and robust throughout the genome,^18–20^ and modern PCR-free library preparations minimize the hazard of capture and PCR-based artifacts. This uniformity of signal enables sample-specific depth normalization that eliminates the need for batch processing.^10,21^ Furthermore, coverage of the noncoding genome allows for increased resolution to detect small CNVs, more accurate estimation of variant boundaries, and in many cases direct evidence for the underlying DNA rearrangement via observation of paired sequencing read alignments.^12,22^ Previous work has shown that GS-based CNV detection can be successfully employed to examine variation in gene dosage and its contribution to genetic diversity,^23^ identify selective signatures of copy-number variation,^12^ and to serve as a potential element of a diagnostic test.^24,25^

Here we describe the technical feasibility assessment and validation of a CNV calling pipeline implemented within an existing clinical genome sequencing (cGS) test. The results demonstrate favorable cGS performance compared with a microarray-based clinical diagnostic test, with a minimal increase in interpretation burden. A retrospective assessment of patients with rare, undiagnosed, and genetic disease (RUGD) profiled with CNV-enabled cGS reveals a wide range of mutations, and suggests that the use of GS as a unified testing platform for genetic disease is possible.

## Materials and methods

### Genome sequencing and CNV calling

DNA samples from cells or patients (see below) were prepared using the Illumina TruSeq PCR-free kit and sequenced on a HiSeq X with paired-end 150-bp reads in the Illumina Clinical Services Laboratory (ICSL, Illumina, San Diego, CA). Data were mapped to the hg19 reference genome with the ISAAC aligner.^26^ The resulting BAM files were analyzed with version 1.3.9 of the Canvas CNV caller^10^ under its germline setting, with modifications to the default calling parameters as follows:

- The circular binary segmentation (CBS) segmentation algorithm was used as opposed to the Haar wavelet–based default in Canvas v1.3.9. This is specified as a parameter on the Canvas command line invocation.
- To limit fragmentation of large CNVs, candidate CNV calls of the same type (e.g., deletion) and copy-number state that were spaced by less than 100 kb were merged into a single call. When such a merge occurred, the magnitude of the gap between segments and any implications on variant interpretation were assessed during manual curation.
- To increase sensitivity in 10–50 kB range, support thresholds for candidate CNVs were dropped to 8 depth bins (a depth bin is defined as a sequence range with an expected 100 reads mapping) in contrast to the default 10 depth bins normally required.
- An automated b-allele-based ploidy correction step was omitted, to limit false negatives. Screening for presence of heterozygous variants in a candidate deletion occurred during manual curation and review.
- Within the CNV calling pipeline, the gray list of filtered regions supplied within Canvas was supplanted with a minimal list of chromosomal segments covering centromeres. The complete gray list was used for call filtering after the full call set is generated (see “CNV region annotation and filtering” below).
- Canvas quality scores were not used as a filter for candidate CNV events.

### CNV truth set generation and sensitivity assessment

Twenty reference samples (Coriell, Camden, NJ) were chosen for validation (Table S1). Among these, 18 samples had known pathogenic CNVs representative of a large size range and inclusive of deletions and copy-number gains, and two samples were included as negative controls. Prior to sequencing and analysis, coordinates for this set of truth CNVs were compiled from descriptions on the Coriell website, reference publications, or previously conducted microarray-based CNV analyses^27^ (Table S1). Note that while all cell lines contain pathogenic CNVs, which established the baseline for our sensitivity analysis, we also examined all other CNVs detected in these samples by either microarray or cGS (see Results).

DNA samples were procured from Coriell and libraries were processed through our standard sequencing and bioinformatics pipeline. In parallel, samples were assessed by an external clinical microarray lab (CombiMatrix Diagnostics, Irvine, CA), which included profiling on an Illumina 850k feature single-nucleotide polymorphism (SNP) array followed by automated CNV calling and manual curation by trained cytogeneticists. One sample failed microarray quality control, resulting in 17 positive control samples for further analysis.

To assess sensitivity, cGS and microarray call sets were compared (in two separate analyses requiring 50% or 75% overlap) with reference calls (Table 1, Table S2, see also Supplemental Note). For false negative calls or calls with only partial overlap with the reference call, visualization of depth and microarray data were employed to assess the accuracy of the call boundaries or identify discrepancies of cGS-based boundaries with the vendor supplied CNV annotation (Results and Supplemental Note).

**Table 1:** Summary of sensitivity of cGS and clinical microarrays to annotated CNVs in cell lines

Event	Size	Coriell events	Called by array^a^	Called by cGS^a^
Loss	10–50 kb	5	3 (+1)	4
	50–100 kb	1	1	1
	100–500 kb	9	3 (+1)	6
	>500 kb	6	6	6
	Overall	21	13 (61%)	17 (80%)
Gain	10–50 kb	3	0	3
	100–500 kb	5	4	4
	>500 kb	7	6	7
	Overall	15	10 (67%)	14 (93%)
All CNV calls		*n* = 36	23 (64%)	31 (86%)

Note that + 1 indicates calls that were not in call set, but recovered in manual review.

*cGS* clinical genome sequencing, *CNV* copy-number variant.

^a^50% overlap.

### Assessment of clinical cGS CNV calling false positive rate

To assess false positive rate, the 17 Coriell cell lines were also profiled on Illumina Infinium Omni 2.5 arrays within the Illumina Clinical Services Laboratory. For each array, raw data were processed within the Illumina GenomeStudio genotyping module and median adjusted logR values were used in downstream analysis. Due to the batch normalization protocol and array resolution limits, we only considered autosomal CNVs detected in a single sample that spanned at least four probes in the array. CNV calls were assessed for array-based confirmation by comparing the median probe depth in the candidate region against a size-matched background on chromosome 1 and an empirical *P* value was calculated. For labeling of putative mosaic CNVs, predicted coverage thresholds of 1.75X and 2.25X were used for gains and losses, respectively.

In addition to microarray confirmation, we also compared cGS-based CNV calls with an external call set derived from a combination of Pacific Biosciences (PacBio) and BioNano DNA data^28^ made on reference genome NA12878. A CNV call was considered validated if there was at least 75% overlap with reference call boundaries. Calls with partial overlap were manually curated to assess possible false positive or partially called reference CNVs resulting in a low overlap (see Supplemental Note). CNVs that were not called in the PacBio  + BioNano data set were manually reviewed for the presence of discordant sequencing reads spanning the boundaries of a deletion or copy-number gain, and the presence of hemizygous and homozygous deletions with similar breakpoints in an independent set of samples from population controls (*n* = 3000).

### Assessment of reproducibility

Sixteen Coriell samples were run on two sequencing lanes each to assess the reproducibility of the cGS/Canvas calling pipeline. To assess reproducibility across distinct sequencing runs, DNA extracted from the 1000 Genomes sample NA12877 was sequenced three times on different days by different sequencing technicians.

### Clinical cohort inclusion criteria

CNV calling and interpretation was deployed as part of the TruGenome Undiagnosed Disease test for patients assessed on or after 2 June 2016. At the time of manuscript preparation, 79 patients had consented for testing with CNV calling included. Patients had a wide range of clinical phenotypes, as well as previous testing ranging from no prior molecular investigations to panels and exome sequencing. The age at the time of testing ranged from 1 to 20 years of age.

For this retrospective study, analysis was conducted on de-identified information under protocol exemption from the Western Institutional Review Board.

### CNV region annotation and filtering

All CNV calls were processed through a series of automated filtering steps to reduce false positives and limit downstream CNV curation to those likely to have medical relevance. CNVs were annotated with overlapping or nearby (<5 kb away) genes using RefSeq gene definitions. The heuristic cutoff of 5 kb was chosen to account for uncertainty in call boundaries as well as include promoter regions without increasing the number of calls passing through to interpretation. Calls with no gene proximal annotation were removed. In addition Canvas provides a set of gray list regions that contains problematic genomic segments as well as common CNVs. After calls were generated, CNVs having greater than 50% of their range spanned by gray list regions were removed.

### Population frequency annotation and filtering

CNV population frequency was estimated using an internal database 3000 samples sequenced in ICSL. These samples were matched to the clinical pipeline sequencing chemistry (PCR-free 2× 150-bpgenome sequencing) and bioinformatic data processing (ISAAC aligner). Additionally, sequencing coverage was normalized to match our clinical implementation of the Canvas CNV caller. To allow for efficient storage and recovery of data across many samples, binned sequencing depth data (an intermediate output of Canvas) was mapped to a fixed 300-bp uniform coordinate system. Due to uncertainty in the boundaries of many CNV calls, a heuristic calculation of CNV population frequency was implemented that includes (1) interrogation of the aggregate sequencing depth data across 3000 genomes for the genomic interval defined by the CNV boundaries, (2) mean depth calculation for each sample, and (3) assessment of the fraction of the population with depth consistent with the proband GAIN or LOSS status. Note that for events on a sex chromosome, only samples with the same gender from the population are queried.

For each clinical case CNVs with a population frequency higher than 10% (~5% allele frequency) are removed from the interpretation call set.

### Interrogation of CNVs indicative of large structural rearrangements

Sequencing reads adjacent to CNVs can provide evidence of complex chromosomal rearrangements. For CNVs indicative of large SVs—including terminal chromosomal deletions, large tandem duplications, and breaks spanning nonhomologous chromosomes—the Manta SV caller^29^ (version 0.29.3) was employed. This enabled breakpoint linkage across multiple CNVs, and provided evidence of the insertion of duplicated sequence. Reassembled breakpoints were visualized by realignment of sequencing reads using the SVViz program.^30^

### CNV phasing

Where possible, phasing was assessed by genotyping parental haplotypes using depth information, or using inheritance patterns of small variants when no evidence of a depth change is present in a parent (e.g., de novo CNVs or duo cases where there is no evidence of a CNV in the sequenced parent).

The de novo CNV phasing algorithm first constructs prior state probabilities given the genotypes of the parents and the known copy number of the proband. Given the prior probabilities of each transition, the model likelihood is computed for all possible inheritance assumptions.

For example, at a haploid (copy number 1) site where the mother is heterozygous (0/1) and the father is homozygous reference (0/0) for a given SNV:

- under the assumption that the CNV is inherited from the father, the probability of a REF or haploid SNV call in the proband are both 50%;
- under the assumption that the CNV is inherited from the mother, the probability of a REF or haploid SNV call in the proband are 100% and 0% respectively.

Probabilities for all inherence assumptions are calculated across all SNVs within the target region, and the most likely inheritance model is selected. For details of inheritance models and examples, see Supplemental Note.

### Visualization of genome-wide depth and B-allele frequency

The depth profile output of the Canvas CNV calling pipeline is visualized for across each chromosome as a “digital karyogram” (e.g., see case P17 below). This serves DNA extraction uniformity quality control metric and cGS data quality, and enables rapid identification of large chromosomal events such as large duplications and deletions, trisomies, and other aneuploidies and uniparental (iso)disomy. To visualize coverage, normalized depth data is filtered against a set of positions known to be variable in population samples, grouped into 100-kb genomic segments, and the depth distribution within each group is visualized to create a heatmap. For b-allele frequency, a similar protocol is followed using larger 500-kb bins and visualized separately.

## Results

### GS CNV calling performance

An assessment of 17 reference samples with reported pathogenic CNVs (Table S1) demonstrated that cGS had greater sensitivity to detect known CNVs compared with microarrays (86% vs. 64%, McNemar’s test *P* *<* 0.01, Methods, Table 1, Table S2) with the greatest difference in smaller (<50 kb) events (Table 1). For the five truth set CNVs not recovered by cGS, manual inspection of sequencing and genotyping arrays did not support a CNV in these regions (Methods, Supplemental Note).

To assess the cGS false positive rate, 80 deletions and 58 copy-number gains across the 17 Coriell samples were assessed for support in corresponding Illumina Infinium Omni 2.5 microarray data (Methods, Figure S1). For 77/80 (96%) of deletions the median array probe signal intensity fell below the expected value for a diploid CNV call, and 36/58 (62%) of gains showed above average signal intensity (empirical *P* < 0.01 for all calls, based on size-matched distribution of background probes). The majority of unconfirmed CNVs had depth indicative of a putative mosaic copy-number variants (3/3 deletions and 19/22 gains; see Methods and Figure S1).

To further assess sensitivity, comparisons were made against a reference set of CNVs for NA12878 from Haraksingh et al.^31^ In this work, 17 available array designs were systematically compared using NA12878. Using these call sets, cGS recovered 126/227 (56%) of all reference CNVs (*n* = 227, size 10 kb+, 50% overlap threshold), which was higher than any array design. Furthermore, cGS recovered 93% of reference CNVs called on at least two different array technologies.

To assess specificity, we compared the 93 deletions called by cGS with Canvas in NA12878 with a data set derived from two alternative DNA sequencing technologies (PacBio and BioNano, see Methods),^28^ which confirmed 48/93 deletions. Among the remaining calls, nine were supported by the presence of discordant sequencing reads or evidence of Mendelian inheritance across a population of samples, leaving 39% (36/93) unvalidated by alternative sequencing technology. We suspect that this set of unvalidated calls includes both false positives and well as suspected true calls without external support.

To assess reproducibility, the CNV reference panel was sequenced across two sequencing lanes each, with 86% (2696/3135) of CNVs called on the first lane recovered in the second using a 75% overlap threshold. Additionally, DNA extracted from NA12877 was sequenced three times on different days by different technicians. Using the first run as a reference, 87% and 86% percent of calls (*n* = 187, 75% overlap threshold) were replicated on the second and third runs, with 82% replicating across both trials (odds-ratio 3, *P* < 10^−10^).

We found that the majority of nonreproduced CNVs and putative false positives could be addressed with minimal heuristic filters. Specifically, removal of CNVs in regions with variable data quality, elimination of CNVs commonly seen in a background population (10% frequency cutoff, *n* = 3000 genomes, see Methods, Figures S2–S3), and scrutiny of putative mosaics (read depth suggesting a noninteger copy number) improved CNV data quality and reduced our interpretation burden. For an average case these steps removed ~90% of CNVs yielding an average of 10 CNVs for interpretation, with one additional CNV removed by manual review. Given these findings, these heuristics were deployed as a component of the cGS pipeline.

### Retrospective case analysis

Seventy-nine clinical cases were processed through the validated cGS CNV pipeline between 2 June 2016 and 19 April 2017 and subjected to automated quality control, filtering, annotation, and visualization (Figures S2–S4, Methods). Passing variants were manually assessed for quality (Figure S4) and interpreted using in-depth literature curation protocol and in accordance with the American College of Medical Genetics and Genomics (ACMG) guidelines for variant classification^32,33^ (Methods, Figure S3a–d). Clinically relevant losses or gains greater than 10 kb were reported, although the clinical test definition made limited claims of sensitivity in the 10–20 kb size range due to the limited availability of truth data in this range. On average, we reported three benign, three variant of uncertain significance (VUS)–likely benign, and four VUS CNVs per case (Figure S3e–g). In 15% (11/79) of cases, we reported variants with pathogenic or uncertain significance–likely pathogenic classifications across a diverse set of patient phenotypes (Table 2). All reported variants were confirmed by external orthogonal testing or Illumina Infinium Omni 2.5 microarrays run within the clinical lab (Supplemental Note).

**Table 2:** Summary of clinically relevant CNVs

ID	Chromosome	Event	Pertinent patient phenotypes^a^
P1^b^	Xq11.2	55 kb de novo loss including first three exons of *ZC4H2*	Arthrogryposis, limited mobility of the proximal muscles of the shoulders and lower extremities, spastic paraparesis, abnormal myelination on MRI, bilateral ulnar deviation and shortened deformed fingers, reactive airway disease, dysarthria, and global developmental delay
P2^b^	22q11.21	434 kb de novo gain	History of multiple bone fractures, hypotonia, delayed motor skills, strabismus, hypermobility, flat feet, and joint pain **Note:** In addition to the CNV identified, a missense variant in *WNT1* was identified providing an explanation for bone fragility and other associated phenotypes
P3^bc^	Xq13.1	9 kb de novo loss encompassing exon 11 of *HDAC8*	Delayed motor milestones, hypotonia, intrauterine and postnatal growth retardation, and dysmorphic features suggestive of Cornelia de Lange syndrome
P4^b^	2p11.2	228 kb maternally inherited gain encompassing *REEP1*	Demyelinating disease observed on MRI, decreased temperature sensation to cold in the distal lower extremities, decreased sensation to vibration in the distal lower extremities, decreased reflexes, mild dysmetria, and bilateral pes cavus **Note:** This CNV was inherited from the proband’s mother who was noted to be similarly affected
P5	16p11.2	223 kb tandem duplication on *SH2B1*	Connective tissue disorder and hypermobile joints, speech delay, speech apraxia, autism, dysmorphic facial features, recent weight loss, short stature, and an abnormal response to traumatic pain **Note**: Finding likely explains diagnosis of autism and related clinical findings, but there is no evidence to suggest that this patient’s connective tissue disorder is related to this CNV
P6	2q37.2→2qter, 3q29→3qter	Mosaic unbalanced translocation	Dysmorphic facial features and congenital anomalies, with scaphocephaly, prominent metopic ridge, hypoplastic supraorbital ridge, high arched eyebrows, epicanthus inversus, short upslanting palpebral fissures, ptosis, blepharophimosis, narrow upper lip, mild micrognathia or retrognathia, midline cleft palate, patent ductus arteriosus, palmar crease abnormalities, tapered fingers, and hypoplastic nails; notable other phenotypic features include failure to thrive, developmental delay, intellectual disability, profuse sweating during feedings, and tachycardia
P7	2pter→2p25.3, 16q23.3→16qter	Unbalanced translocation	Microcephaly and severe intellectual disability, with no speech and behavioral problems including repetitive, aggressive, and self-abusive behavior; patient described as having sleep difficulties, ataxic walking, and dysmorphic facial features including downslanting palpebral fissures, full lips, frontal upsweep, ptosis, strabismus, and dental crowding
P8	19q13.11-12	1.7 MB de novo deletion	Progressive dystonia, prematurity (born at 28 weeks), dysarthria/anarthria, tongue dyskinesia, microcephaly, abnormal ocular movements, intellectual disability, some repetitive obsessive behaviors, and pyramidal tract signs on MRI; nonverbal and does not walk or eat independently; described as thin-appearing
P9	18p	Tetrasomy 18p	Severe global developmental delay, nonverbal, ataxia, feeding difficulties, strabismus, aggressive behavior, dysmorphic features including occipital plagiocephaly, downslanting short palpebral fissures, low-set posteriorly rotated malformed small ears, smooth philtrum, mild prognathism, bilateral camptodactyly in the 3rd, 4th, and 5th fingers with absent distal interphalangeal creases, hypoplastic thenar eminences, and a right single transverse palmar crease; facial paralysis as an infant and asymmetric crying face
P10	8p23.1	5.1 Mb gain (unknown de novo or inherited)	Intermittent rash; telangiectasia; acroparesthesia; numbness, pain, and swelling of extremities; joint pain; facial flushing; and headaches; CT scan revealed hypoperfusion in the left parietal lobe relating to ischemia; he also has hypertrophic cardiomyopathy, bradycardia, expressive speech delay, and a learning disability
P11	6q22.1-31, 6q23.1, 11p15.4-3	Multiple large deletions on 6q, inserted duplication of 11p into chr17	Growth deficiency, microcephaly, intellectual disability with no speech, hyperactivity, large bulbous nose, epicanthal folds, short philtrum, small mandible, seizures; family history is pertinent for two maternal uncles who have little or no speech
P12	14q32.2	23 kb de novo mosaic deletion to the promoter of *MEG3*	Developmental delay, speech delay, behavioral difficulties, neonatal respiratory and feeding difficulties, hyperextensible and buckling phalanges, bilateral hallux valgus, and dysmorphic features including full cheeks, myopathic facies, prognathism, thick pinnas, strabismus, short forehead, bifrontal narrowing, midface hypoplasia, and anteverted nares
P13	15q11.2	604 kb maternally inherited deletion of BP1–BP2 in the Burnside–Butler susceptibility locus	Episodes of ataxia, cyanosis, memory disturbance, speech difficulty, emesis, and severe pain in arms and legs, easy fatigue, constipation alternating with diarrhea, possibly due to intestinal dysmotility, as well as general abdominal distension and possible intussusception; ventricular tachycardia, frequent respiratory infections and rashes, and possible small fiber neuropathy were also noted; differential diagnoses include dysautonomias, mitochondrial disorders, energy depletion syndromes, and mast cell abnormalities
P14	16p13.11	1.6 Mb gain, unknown inheritance	Congenital inflammatory myopathy, hypotonia, muscle pain, absent reflexes, and motor delay; a muscle biopsy showed necrosis and paleness of sarcoplasma with eosinophilia, multiple vacuoles, and inflammatory infiltrate **Note:** This CNV was reported as a pathogenic incidental finding for 16p13.11 microduplication syndrome
P15	7p22.1	749 kb deletion in *PSM2*	Phenotype not applicable as CNV was discovered upon Secondary Findings analysis
P16	chr21	Trisomy 21	Clinical diagnosis of Down syndrome (confirmed by karyotype). Additionally, patient is reported to have phenotypic features that are not consistent with a diagnosis of trisomy 21, including hypotonia that is more marked than expected, dermal ridge patterns with more arches than are typical, a small phallus, infantile spasms that are controlled on medication, a skeletal and long myopathic face, lumbar lordosis, scapular winging, tapered calves, absent reflexes, ptosis, and a lurching pelvis when walking. The patient is unable to walk without a walker. Family history is notable for nemaline myopathy. The patient’s mother’s phenotype includes weakness in childhood and a muscle biopsy revealing nemaline rods, consistent with a diagnosis of nemaline myopathy. She could not run or jump as a child, although her clinical presentation improved over time. Currently, she is reported to have few residual features although she cannot run. **Note:** CNV analysis revealed trisomy 21; in addition, a maternally inherited variant classified as likely pathogenic in *ACTA1* was identified in both the proband and mother
P17^c^	chr14	Mosaic trisomy 14	Developmental delay, ocular colobomas, low-set posteriorly rotated ears, hypomelanosis of Ito, solitary kidney, atrial and ventricular septal defects
P18^c^	chr15	UPD15, paternal	Global developmental delay with language delay, strabismus, coxa vulga, genu valgum, pes planus, low-set cupped ears with attached lobe, broad palate with alveolar ridge, short neck, inverted nipples, truncal obesity, broad-based ataxic gait, hypotonia, and lumbar lordosis
P19^b,c^	chr16	UPD 16, paternal	Hypotonia, developmental delay, diffuse pachygyria with leukoencephalomalacia **Note:** Paternally inherited UPD 16 was considered an incidental finding for this patient as there is no evidence linking paternal UPD 16 in association with disease

Note that these variants are not used in the aggregate statistics reported here.

*CNV* copy-number variant, *MRI* magnetic resonance image, *UPD* uniparental disomy.

^a^Patient phenotypic data were provided to Illumina Clinical Services Laboratory by the ordering physician via the completed test requisition form and accompanying medical notes.

^b^Sample sequenced in prevalidation test development cohort. Note that these samples are not used in any reported aggregate statistics.

^c^Variant is outside of the clinical test definition but was observed in a development pipeline or as an incidental finding.

**Fig. 1:**
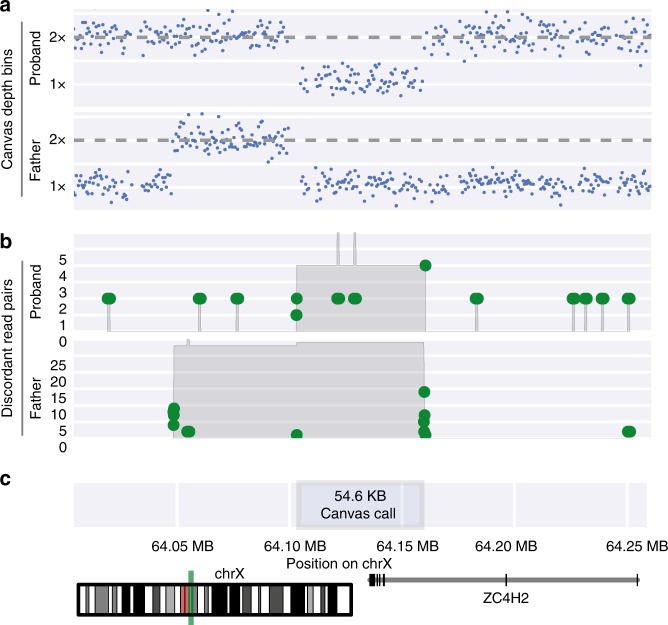
***ZC4H2*** **de novo deletion in case P1.** **a** Normalized sequencing depth for proband and her father. **b** Location of discordant read-pairs (>1000 bp insert size), where green dots represent the location of paired ends in a discordant read-pair, the gray shaded area represents the total number of discordant read-pairs spanning a given genomic segments. **c** Annotation of the original Canvas call boundaries as well as the location of the copy-number variant (CNV) on chromosome X.

We found that the combination of depth-based CNV calling and the utilization of discordant read-pair information can enable deconvolution of complex rearrangements. In one example, family-based CNV analysis of case P1 identified a 55-kb de novo deletion of the first three exons of *ZC4H2* on the paternal X chromosome (Fig. 1), consistent with Wieacker–Wolff syndrome (Table 2). CNV boundary analysis (Methods) identified evidence for a tandem duplication in the proband’s father, sharing a breakpoint with the deletion in the proband (Fig. 1b), while read depth information from the father showed a copy-number gain directly upstream of the de novo deletion in the proband (Fig. 1a). Taken together these data likely indicate a multistage repair mechanism contributing to the copy-number loss in the proband.

Similarly, case P11 harbored multiple CNVs indicative of a large chromosomal disruption event^34^ including 15.5-MB and 2.5-MB deletions on 6q along with a 2-MB copy-number gain on 11p (Figure S5a, Table 2). Inspection of SVs near these events supported the presence of simple deletions as opposed to more complex events such as translocations or inversions (Figure S5b); however, such complex SVs cannot be definitively ruled out. In contrast, SVs near both boundaries of the 11p gain indicated nonhomologous chromosome junctions, providing evidence of an insertion of this duplicated DNA segment into 17q21.3 (Figure S5c).

**Fig. 2:**
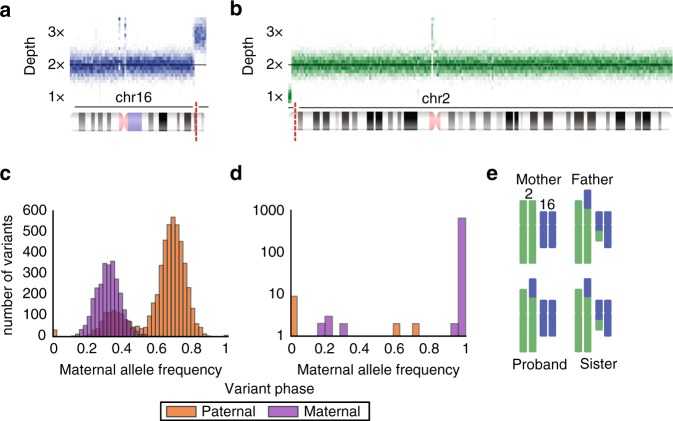
**Case P7: derivative chromosome inherited from a balanced translocation in a parent.** **a**, **b** Sequencing depth support for **a** duplication on 16q and **b** deletion on 2p. Slices in the image represent distribution of normalized sequencing depth across 100-kb genomic intervals. **c**, **d** Distribution of maternal allele frequency for all phased variants in copy number altered regions corresponding to **c** 16q gain and **d** 2p loss. Note that variant frequency distributions are colored by the parent of origin as determined by trio phasing. **e** Summary of split and discordant sequencing read evidence for recombinant chromosomes at copy-number variant (CNV) breakpoints.

CNV analysis of case P7 (Table 2) identified a 7-MB de novo terminal duplication on 16q and a 3-MB de novo terminal deletion on 2p (Fig. 2a, b). Analysis of variant allele frequencies overlapping these two CNVs phased both alterations to the paternal chromosome (Fig. 2c, d, Methods, Supplemental Note). Subsequent analysis of sequencing reads provided evidence for a balanced translocation in the father as well as the unaffected sister, while the proband had support for an unbalanced translocation (Fig. 2e, Figure S6, Methods).

In case P6 we observed a similar unbalanced translocation with a nonhomologous break-end linking the centromeric breakpoints of the two large terminal CNVs. Further inspection of copy-number depth as well as variant allele frequencies indicated that the CNVs were likely mosaic in the blood, with both events having similar estimated purity of 60–64% (Figure S7), which was independently confirmed at 63% by clinical microarray. Taken together these events suggest the presence of a mosaic unbalanced translocation in the affected proband, a rare event that has been previously noted, but for which the mechanism of formation is still unclear.^35^

**Fig. 3:**
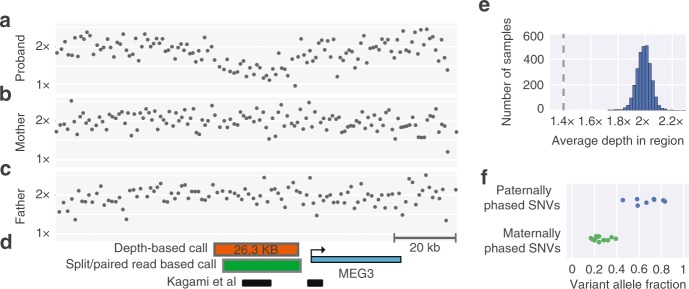
**Mosaic 14q32.2 26-kb microdeletion.** **a–c** Normalized depth across pedigree sequenced for subject P12. Shown here is the genomic region between 101.21 MB and 101.34 MB on chromosome 14 (hg19 coordinates). **d** Annotations for the genomic region. The orange box represents the Canvas CNV call boundaries, the green box represents breakpoint assembled coordinates of the deletion from the Manta SV caller, the black lines represent subjects from Kagami et al.^36^ with deletions in this region, the blue box represents the gene boundaries of the imprinted gene *MEG3*. **e** Average depth across the region of the CNV call for samples across an internal reference population; depth for the proband is indicated with a horizontal dashed line. **f** Variant allele frequency for the SNVs within the deleted region. *CNV* copy-number variant, *SNV* single-nucleotide variant, *SV* structural variant.

In case P12, CNV analysis identified a 23-kb de novo deletion 4.5 kb upstream of *MEG3* completely overlapping the IG-DMR region of 14q32.2 previously implicated in Kagami–Ogata syndrome^36^ (Fig. 3). Further analysis indicated that the CNV was likely mosaic, present in about 50% of cells, and that the deletion phased to the maternal allele, consistent with the paternal imprinting mechanism of Kagami–Ogata^36^ (Fig. 3e, f). In this case, the mosaic deletion passed standard CNV analysis and was annotated as mosaic during manual curation. In contrast, in case P17, we identified a mosaic trisomy of chromosome 14 via a genome-wide visualization (Figure S8, Methods). While this mosaic variant was identified outside of our clinically validated pipeline, the variant was sent out for external clinical microarray testing, which confirmed the CNV and estimated its purity at 51%, compared with 47% as estimated by cGS.

## Discussion

Here we report the development and deployment of CNV detection as a component of a clinical genome sequencing test for patients with a suspected genetic disease. Overall, we found that cGS CNV detection is at least as sensitive as microarrays (Table 1, Supplemental Note), with one analysis indicating that cGS is more sensitive than any one of 17 microarray platforms recently assessed.^31^ Our data indicate that the likelihood of false positives is low, but to address the possibility that favoring sensitivity has increased our false positive detection rate, we developed a stringent filtering and curation protocol (Methods, Figure S2-4). This relies on our ability to annotate population frequency across a set of more than 3000 PCR-free genomes (Figure S3d), and to visualize the CNV to assess the underlying data quality (Figure S4). Additionally, and consistent with current microarray practices, we also employ external databases of benign and pathogenic CNVs,^37,38^ internal aggregate data, and previously curated variants to assess the analytical validity of a call and provide a variant classification.^32^ The CNV calling and curation methods described here do not rely on bulk data processing or analysis (i.e., batching of samples), allowing for ingestion and interpretation of one family at a time. Furthermore, these methods are suited to exploit future increases in sequencing coverage that will result in an increase in resolution to call small CNVs, allowing for test improvement with minimal modifications to the sample preparation or bioinformatic pipelines.

A retrospective analysis of the first 79 cases assessed with cGS CNV calling revealed that 15% had clinically significant CNV findings. This included variants that spanned from 23 kb to trisomies and unbalanced translocation events that were disambiguated using anomalous short-read support and interrogation of small variant phasing. We note that this cohort represents a sampling of families from diverse geographic and socioeconomic backgrounds, some of whom had unclear or undocumented prior genetic testing. Future randomized control trials will be best placed to make systematic assessments of cGS performance compared with standard of care, but this study has nonetheless demonstrated that the addition of CNV calling to a currently deployed small variant calling pipeline is likely to improve diagnostic efficacy in a pediatric genetic disease population.

Indeed, in families with previous genetic testing, cGS may still identify new variants and provide a diagnosis. For example, in the case of a child (P14, Table 2) who had a previous negative clinical exome, cGS was able to identify a pathogenic 1.7-MB deletion indicative of 16p13.11 microdeletion syndrome. In subject P16 (Table 2) we observed trisomy 21 in the subject consistent with a preexisting Down syndrome diagnosis, but were also able to identify a likely pathogenic SNV within the *ACTA1* gene conferring the additional diagnosis of an inherited nemaline myopathy. Finally in P12, we observed a 26-kb deletion in noncoding region upstream of a long noncoding RNA (*MEG3*), which may not have been observed by some commercial microarrays or by exome sequencing.

As sequencing costs continue to decrease, the use of a genome diagnostic as a first-line test may become more likely—especially if additional test elements can be implemented that replicate, or improve upon, the current molecular test repertoire. In addition to the CNV calling described here, future improvements are likely to include the detection of small copy-number variants between 50 bp and 10 kb and the systematic detection of mosaic CNVs, especially for large copy-number variants where cGS has sufficient data to detect low purity alterations.^39^ We further anticipate that the employment of specialized variant callers will allow for the disambiguation of complex variant types, including those associated with spinal muscular atrophy^40^ and repeat expansions.^9^

Overall, the data presented here indicate that CNV calling from cGS is robust and can benefit patients with a suspected genetic disease. Further algorithmic improvements, and the availability of large PCR-free genome reference sets, are likely to further increase both the sensitivity and the specificity of the assay and improve its diagnostic efficacy.

## Electronic supplementary material

Supplementary Information
