# Supplementary material for: Copy-number variants in clinical genome sequencing: deployment and interpretation for rare and undiagnosed disease
Source: Genet Med. 2018 Oct 8;21(5):1121–30. doi: 10.1038/s41436-018-0295-y (PMC6752263; doi:10.1038/s41436-018-0295-y)
Supplement: Supplementary file 1 — Supplementary Information [file 41436_2018_295_MOESM1_ESM.pdf]

Supplementary data accompanying the manuscript:

Copy number variants in clinical genome sequencing: deployment and interpretation for rare and undiagnosed disease

Andrew M Gross PhD[1], Subramanian S. Ajay PhD[1], Vani Rajan MS[1], Carolyn Brown CGC[1], Krista Bluske PhD[1], Nicole J Burns MS[1], Aditi Chawla PhD[1], Alison J Coffey PhD[1], Alka Malhotra PhD[1], Alicia Scocchia MS CGC[1], Erin Thorpe MS CGC[1], Natasa Dzidic MS[2], Karine Hovanes PhD FACMG[2], Trilochan Sahoo MD FACMG[2], Egor Dolzhenko PhD[1], Bryan Lajoie PhD[1], Amirah Khouzam MS CGC[3], Shimul Chowdhury PhD FACMG[4], John Belmont MD PhD[1], Eric Roller PhD[1], Sergii Ivakhno PhD[5], Stephen Tanner PhD[1], Julia McEachern PA MHS[1], Tina Hambuch PhD FACMG[3], Michael Eberle PhD[1], R Tanner Hagelstrom PhD FACMG[1], David R Bentley PhD[5], Denise L Perry MS CGC[1] and Ryan J Taft PhD[1,*]

[1] Illumina Inc., 5200 Illumina Way, San Diego, CA, USA

[2] CombiMatrix (currently Invitae), Irvine, CA, USA

[3] Invitae Corporation, San Francisco, CA, USA

[4] Rady Children's Institute for Genomic Medicine and Rady Children's Hospital

[5] Illumina Cambridge Ltd., Chesterford Research Park, Little Chesterford, UK

Table of Contents

Supplemental Figures 3

Figure S1. Inspection of Coriell GS CNVs in microarray data. 3
Figure S2. CNV filtering pipeline. 4
Figure S3. Summary of manual curation and variant annotation across the CNV clinical cohort. 5
Figure S4. Example CNV visualization used for CNV interpretation. 6
Figure S5. Complex rearrangement in subject P11. 8
Figure S6. Read support for unbalanced translocation in subject P7. 9
Figure S7. Depth at CNV breakpoints for a mosaic unbalanced translocation in subject P6. 10
Figure S8. Depth across chromosomes for subject P6 with mosaic trisomy 14. 11

Supplemental Tables 12

Table S1: Coriell reference CNV call coordinates. 12
Table S2. Sensitivity of cGS and clinical microarrays to annotated CNVs in cell-lines. 14
Table S3. Coordinates of reported variants from RUGD cases. 15

Supplemental Note 16

Manual Inspection of NA12878 calls with partial PacBio/BioNano overlap 16
Manual Inspection of Coriell CNV Calls 19

Manual Inspection of Coriell CNV Calls 19
Manual Inspection of Calls with Less Than 75% Reciprocal Overlap 21
Manual Inspection of False Negative Coriell CNV Calls 24

Microarray confirmation of CNVs 28
de novo CNV phasing models 35

Deletion phasing 35
Gain phasing 37

Supplemental References 39

Supplemental Figures

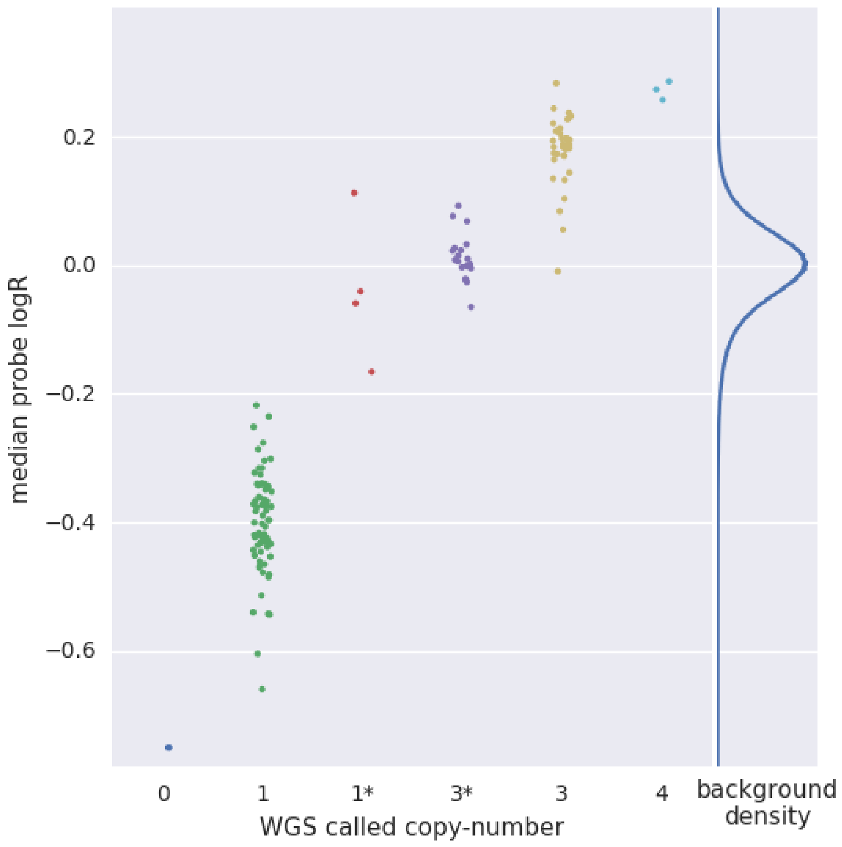

Figure S1. Inspection of Coriell GS CNVs in microarray data. Shown is median microarray probe depth for CNVs called in the GS-Canvas data processing pipeline across a cohort of 17 Coriell cell-lines. The background density on the right of the figure represents the distribution a four probe rolling median across the chip. * indicates putative mosaic copy number states (1.5X-1.75X for deletions and 2.5X-2.75X for copy-number gains).

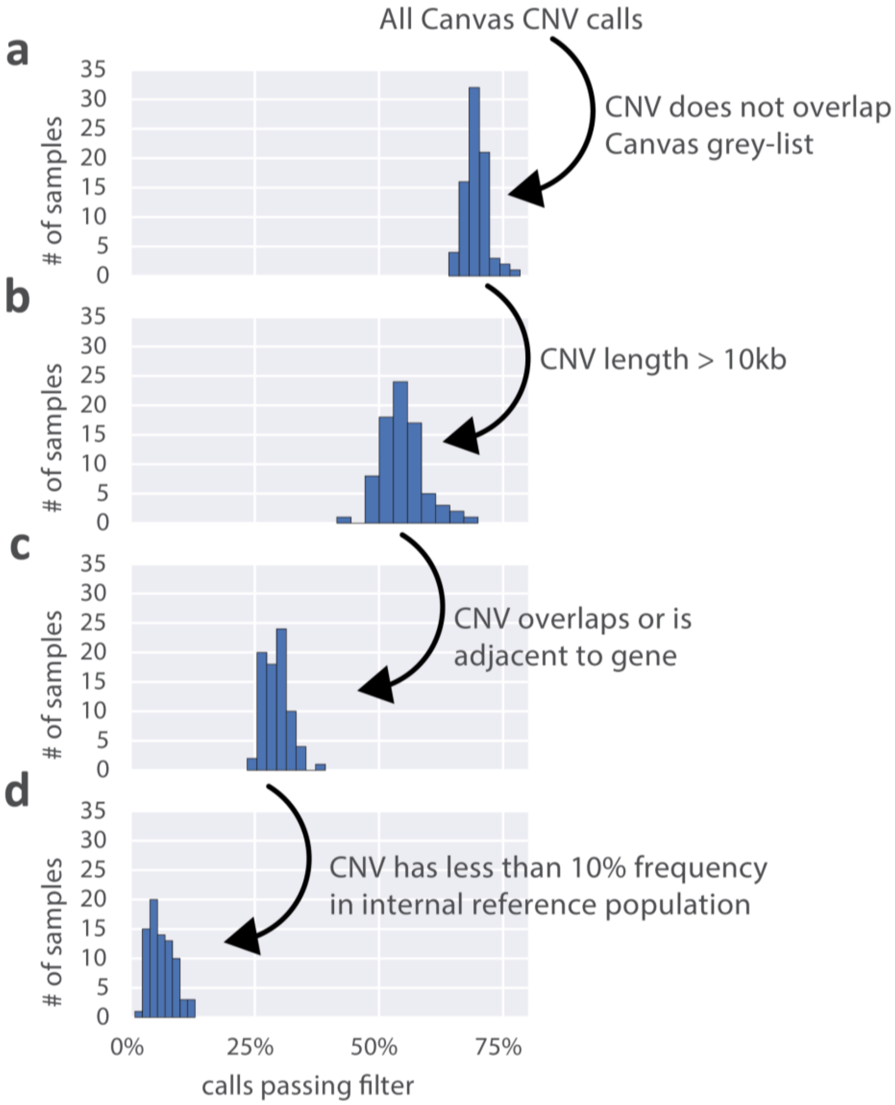

Figure S2. CNV filtering pipeline. During data processing, automated filters are applied to call-sets to limit the number of calls presented to case managers for manual curation. Shown in each panel are the percentage of calls remaining after each filtering step is applied sequentially. Distributions reflect 79 samples assessed for CNVs in the ICSL cohort. For details on individual filters, see **Methods**.

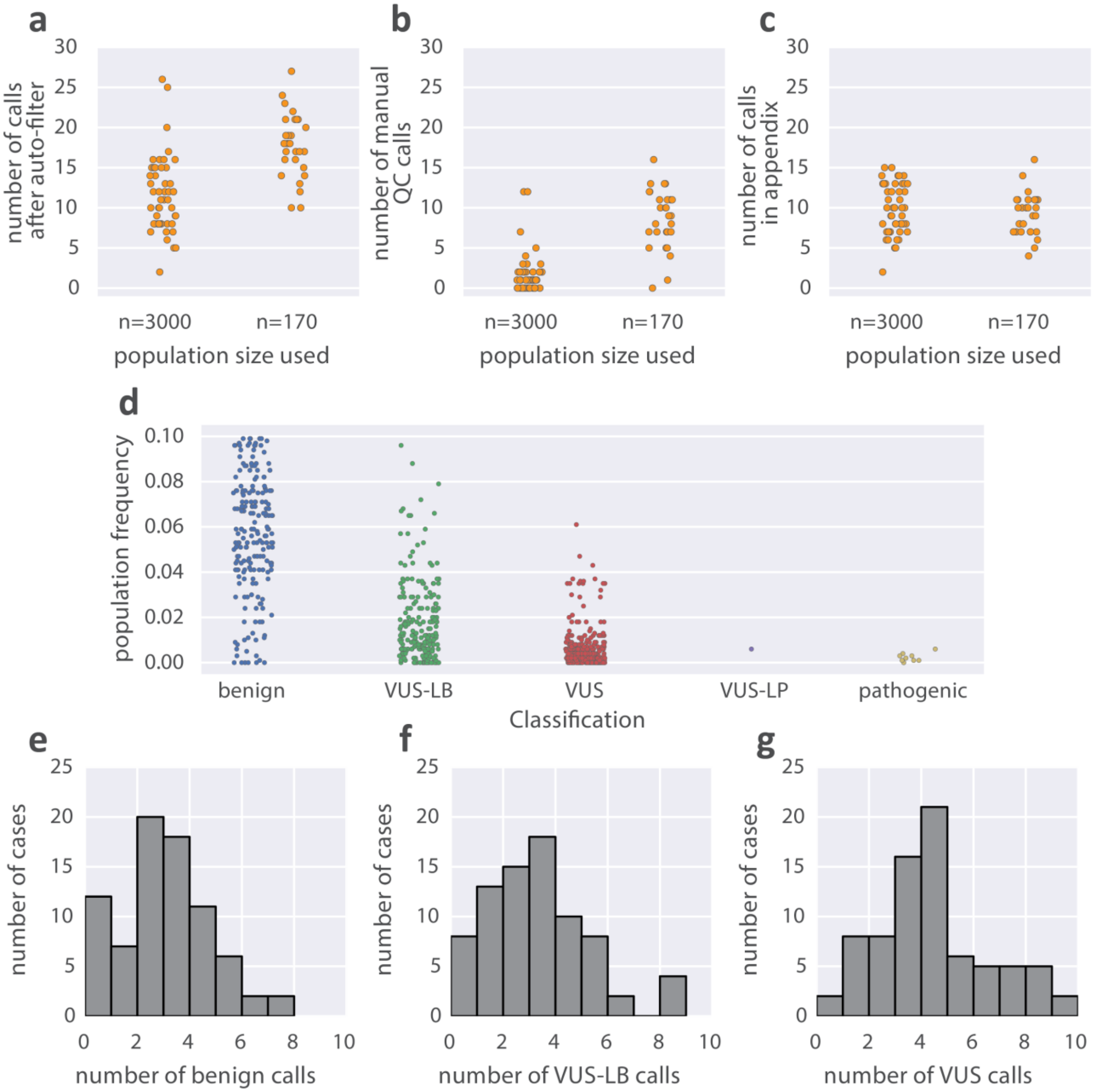

Figure S3. Summary of manual curation and variant annotation across the CNV clinical cohort. (**a-c**) Number of calls passing automated filters (**a**), manually filtered (**b**) and included in a CNV appendix to clinical reports (**c**) broken down by the two populations used for CNV frequency annotation (n=170 was used for the first 28, and n=3000 was used for the remainder). (**d**) Copy number variant population frequency broken down by variant classification post curation. (**e-g**) Breakdown of variant classifications for CNVs included in clinical report appendix across the cohort. In addition there were calls reported as pathogenic in 7 cases, and one likely-pathogenic call reported. VUS - variant of unknown significance; VUS-LB- variant of unknown significance, likely benign.

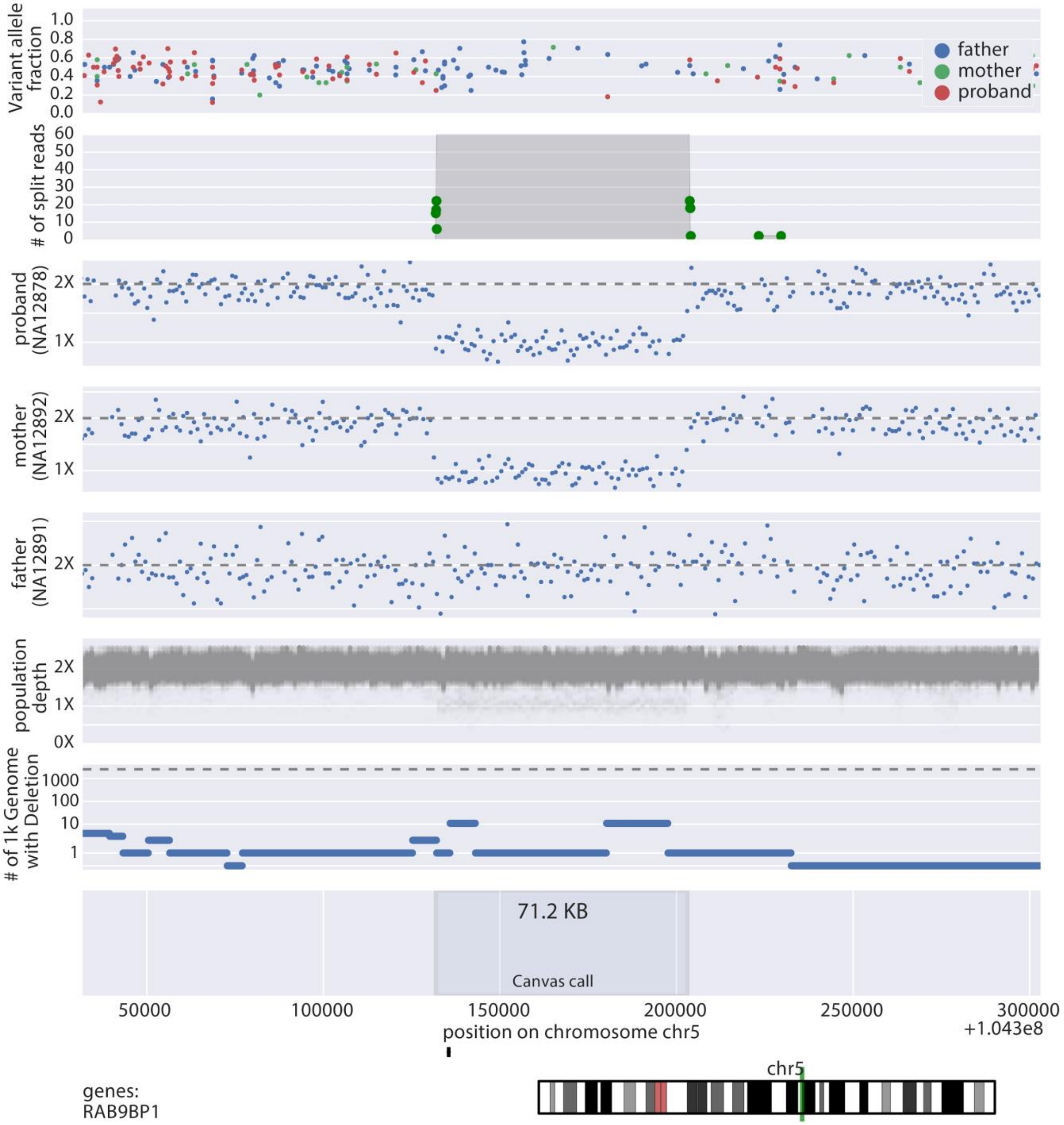

Figure S4. Example CNV visualization used for CNV interpretation. **Variant allele fraction**: read-count ratio of two alleles for all heterozygous SNVs in a region. This fraction should be centered at 1/2 in diploid regions, while for duplications it is expected to be centered around 1/3 and 2/3 as there is an imbalance of alleles. For deletions there should be an absence of heterozygous SNVs due to the presence of only one allele. **Number of Split Reads**: Green dots show the locations of discordant read-pairs, while the height of the grey shaded region indicates the number of discordant reads spanning across a given

region on the genome. Split read information may be used to increase confidence in a CNV call via signatures of deletion or tandem-duplication, but CNVs may have breakpoints in non-unique sequence resulting in an inability to uniquely map reads to the flanks of the CNV. **Read depth**: Normalized read depth across the proband and parents. This allows for evaluation of error modes and evaluation of putative mosaic CNVs. **1000 Genomes Data**: CNV calls the 1000 Genomes Project[1] (GS, 7x coverage) used to identify common deletions. **Population Depth Data**: Normalized coverage for 200 samples selected from our internal population data. This allows for inspection of population trends which may expose artifacts in the read-mapping or data-normalization process leading to a false-positive call. **Chromosome view and overlapping genes**: This field allows the interpreter to view where the event takes place in context of the chromosome and displays the coding sequences of genes that overlap the events so it can be determined if the genes are relevant to the phenotype.

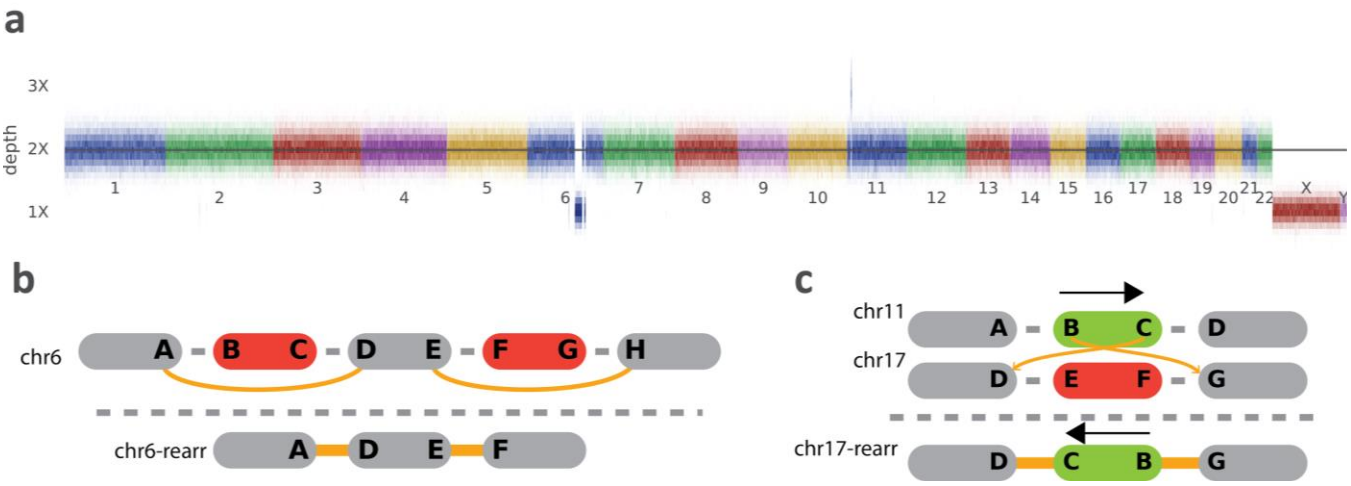

Figure S5. Complex rearrangement in subject P11. **a,** Depth across chromosomes showing two large regions of copy-number loss on chromosome 6 and a copy number gain on chromosome 11. **b,** Schematic of structural rearrangement on chromosome 6 indicating two large deletions in close proximity. **c,** Schematic of structural rearrangement on chromosome 17 indicated a large insertion of genetic material from chromosome 11.

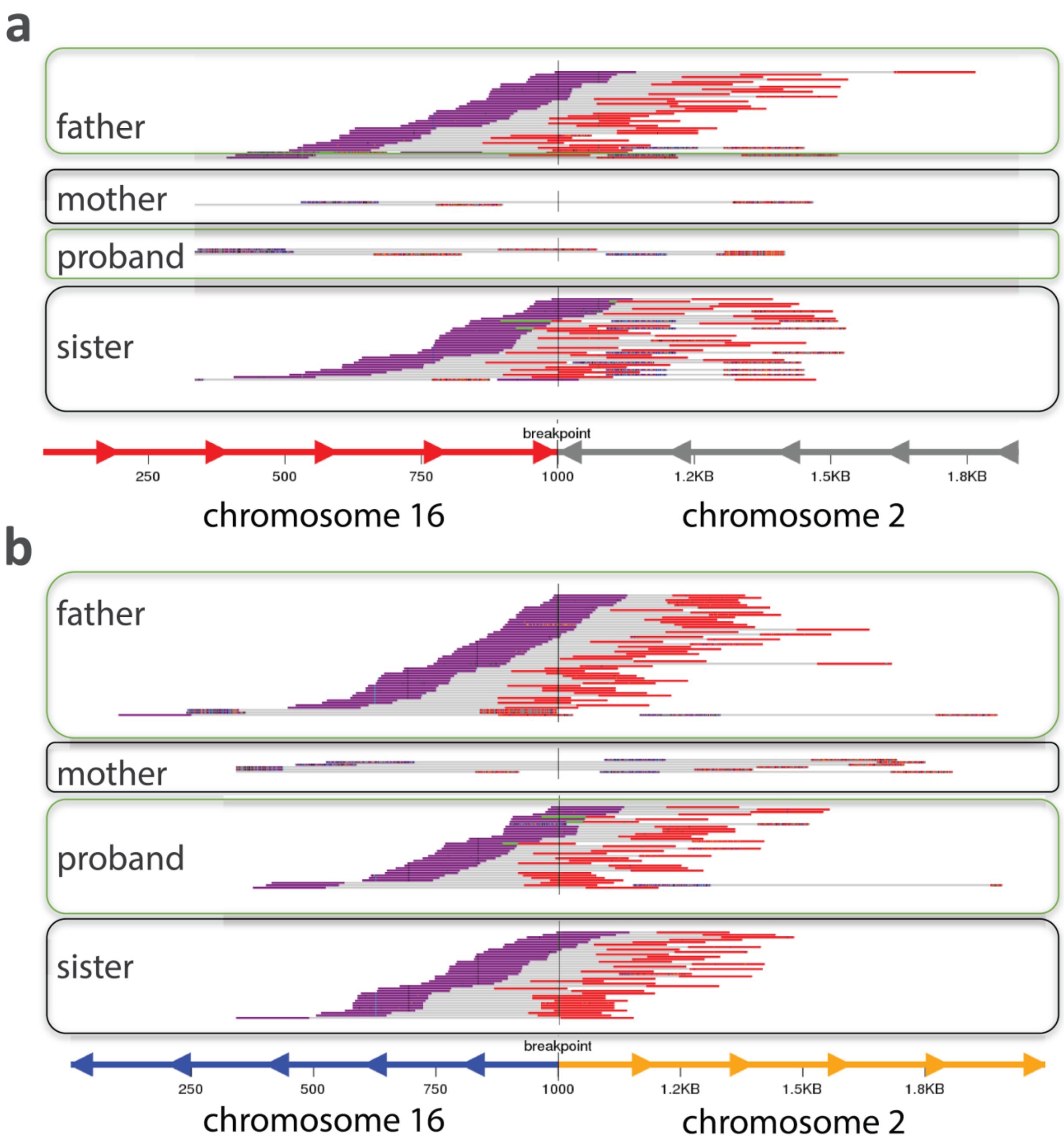

Figure S6. Read support for unbalanced translocation in subject P7. Shown here are modified plots from the svviz graph realignment program[2]. In brief, reads are realigned to normal (not shown) and recombinant (shown here) chromosomes across the pedigree. Purple and red colors represent the first and second reads in the read-pair, respectively, for details on the visualization and realignment methods see Spies et al.[2]. See also **Figure 2**.

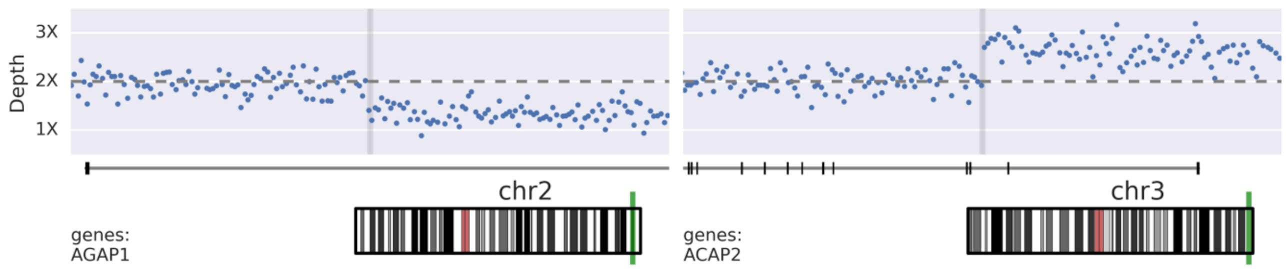

Figure S7. Depth at CNV breakpoints for a mosaic unbalanced translocation in subject P6. Horizontal grey lines correspond to the location of a non-homologous chromosomal break-end uncovered in structural variant analysis.

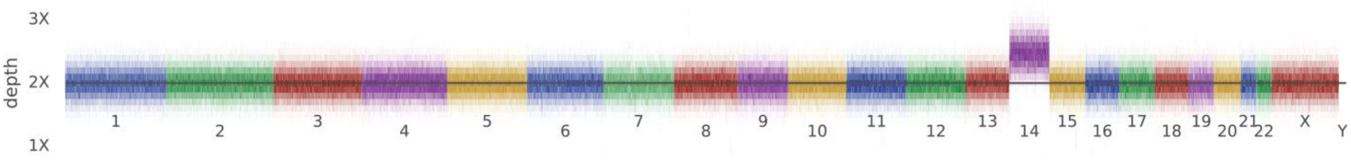

Figure S8. Depth across chromosomes for subject P6 with mosaic trisomy 14. Horizontal line corresponds to diploid copy-number.

Supplemental Tables

Table S1: Coriell reference CNV call coordinates.

subject	CHROM	start	end	gender	CN	event	size (kb)
NA02767	chr21	0	48,129,895	F	3	GAIN	48,130
NA04327	chrX	32,827,464	32,850,164	M	2	GAIN	23
NA04517	chr14	88,399,358	88,429,855	M	0	LOSS	30
NA04520	chr16	2,097,990	2,114,272	F	0	LOSS	16
NA05090	chrX	32,843,154	32,897,248	M	0	LOSS	54
NA06804	chrX	133,607,389	133,620,495	M	2	GAIN	13
NA06804	chrX	133,594,369	133,607,388	M	0	LOSS	13
NA09834	chr19	50,576,403	50,681,994	F	1	LOSS	106
NA09834	chr15	89,456,759	91,764,988	F	1	LOSS	2,308
NA09834	chr9	111,554,622	111,768,395	F	1	LOSS	214
NA09834	chr9	97,860,095	99,648,422	F	1	LOSS	1,788
NA11428	chr3	162,626,559	197,896,005	F	3	GAIN	35,269
NA11428	chr3	162,513,136	162,625,983	F	1	LOSS	113
NA11428	chr3	60,332	5,368,902	F	1	LOSS	5,309
NA11428	chr3	132,724,911	162,513,080	F	3	GAIN	29,788
NA12214	chr17	14,153,961	15,544,134	M	3	GAIN	1,390
NA13554	chr15	25,165,212	25,205,204	M	1	LOSS	40
NA13590	chr17	25,984,092	26,085,108	F	3	GAIN	101
NA13590	chr2	97,886,321	131,157,859	F	4	GAIN	33,272
NA13590	chr2	242,915,453	243,034,674	F	1	LOSS	119
NA13590	chr4	144,842,091	144,943,597	F	1	LOSS	102
NA13590	chr9	33,140,788	33,261,061	F	3	GAIN	120
NA18310	chrX	0	155,270,560	M	2	GAIN	155,271
NA20217	chrX	798,388	998,748	M	0	LOSS	200
NA20217	chrX	585,079	620,146	M	0	LOSS	35
NA20304	chr15	32,458,660	32,876,972	M	1	LOSS	418
NA20304	chr15	20,500,000	22,500,000	M	4	GAIN	2,000
NA21886	chr1	237,231,289	237,441,153	M	3	GAIN	210

NA21886	chr2	100,973,623	101,076,046	M	3	GAIN	102
NA21886	chr18	0	15,000,000	M	1	LOSS	15,000
NA21886	chr18	51,819,089	52,484,051	M	1	LOSS	665
NA21886	chr18	52,612,123	78,015,057	M	1	LOSS	25,403
NA21886	chr4	144,700,854	144,813,390	M	1	LOSS	113
NA21886	chr22	18,781,533	19,006,984	M	3	GAIN	225
NA23127	chrX	32,456,508	32,472,778	M	2	GAIN	16
ND01037	chr6	162,475,207	162,683,557	M	0	LOSS	208

Table S2. Sensitivity of cGS and clinical microarrays to annotated CNVs in cell-lines.

		Coriell Events	Called by Array** (curated)	Called by cGS**
**event**	**size**			
**LOSS**	**10kb-50kb**	5	3 (+1)	4
	**50kb-100kb**	1	1	1
	**100kb-500kb**	9	2 (+1)	5
	**>500kb**	6	6	6
	**Overall**	21	12 (57%)	16 (76%)
**GAIN**	**10kb-50kb**	3	0	2
	**100kb-500kb**	5	4	4
	**>500kb**	7	4	5
	**Overall**	15	8 (53%)	11 (73%)
**All CNV calls**		n=36	20 (56%)	27 (75%)

**75% overlap
Note that +1 indicates calls that were not in call set, but recovered in manual review

Table S3. Coordinates of reported variants from RUGD cases.

			CNV coordinates		SV coordinates		
**ID**	**event**	**chrom**	**start**	**end**	**start**	**end**	**comment**
P1	LOSS	chrX	64104162	64158754			split read evidence but no SV call
P2	GAIN	chr22	21052009	21484438			
P3	LOSS	chrX	71549289	71557651	71549289	71557651	CNV came from development SV pipeline
P4	GAIN	chr2	86283023	86511034	86282714	86510931	
P5	GAIN	chr16	2882331	29047087			
P6	LOSS	chr2	236478472	243048854	236478812		breakpoint links CNVs in unbalanced translocation
P6	GAIN	chr3	195106447	197846145	195105935		breakpoint links CNVs in unbalanced translocation
P7	LOSS	chr2	11314	3033976		3033857	breakpoint links CNVs in unbalanced translocation
P7	GAIN	chr16	82865402	90163542	82865480		breakpoint links CNVs in unbalanced translocation
P8	LOSS	chr19	35223021	36895699	35223614	36896374	
P9	GAIN	chr18	11494	15404287			
P10	GAIN	chr8	7153587	12245784			
P11	LOSS	chr6	109324789	124836619	109325818	124836270	
P11	LOSS	chr6	129969121	132499298	129970203	132499992	
P11	GAIN	chr11	8548056	10497905	8548078	10498608	region inserted into chr17
P11	INS	chr17			41705963	41705972	Genomic material from chr11 inserted here
P12	LOSS	chr14	101261679	101288013	101261679	101288013	
P13	LOSS	chr15	22696624	23301066			
P14	LOSS	chr16	14800000	16400000			approximate boundaries due to low sequence complexity
P15	LOSS	chr7	6027017	6776186			
P16	GAIN	chr21	14596056	48101324			
P17	GAIN	chr14					CNV ID by visualization, boundaries manually assessed
P18	ROH	chr15	23633319	102280298			boundaries assessed by ROH caller
P19	ROH	chr16	80212	90142842			boundaries assessed by ROH caller

Supplemental Note

Manual Inspection of NA12878 calls with partial PacBio/BioNano overlap

In these cases, calls from our GS call-set had partial overlap with the PacBio/BioNano derived calls. We inspected these manually to better understand the discrepancies and assess false-positive or true-positive status.

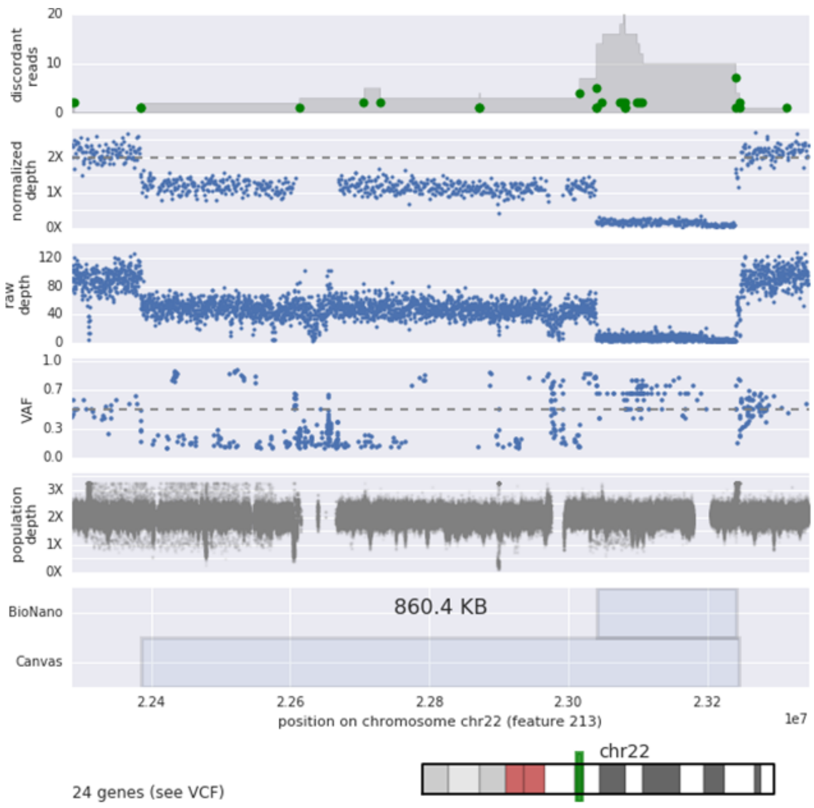

Supplemental Note Figure 1. This is a homozygous deletion flanking a mosaic 22q11 deletion, a likely cell line artifact. Our GS pipeline called this event as a single CNV, whereas the PacBio/BioNano based call-set only contained the homozygous deletion.

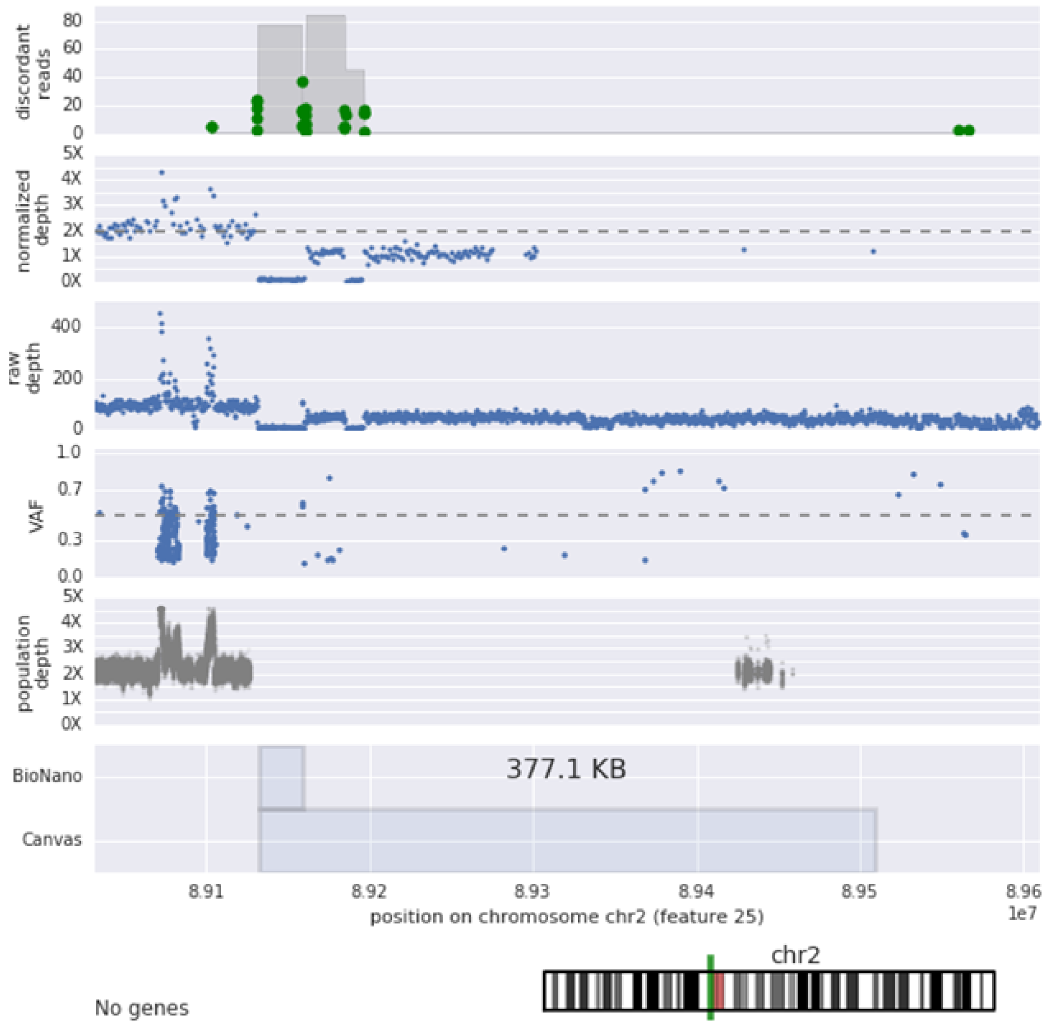

Supplemental Note Figure 2: This CNV is a homozygous deletion followed by a mosaic loss leading up to the centromere of chromosome 2. The homozygous deletion is contained in the PacBio/BioNano call-set, but the mosaic loss is missed or filtered.

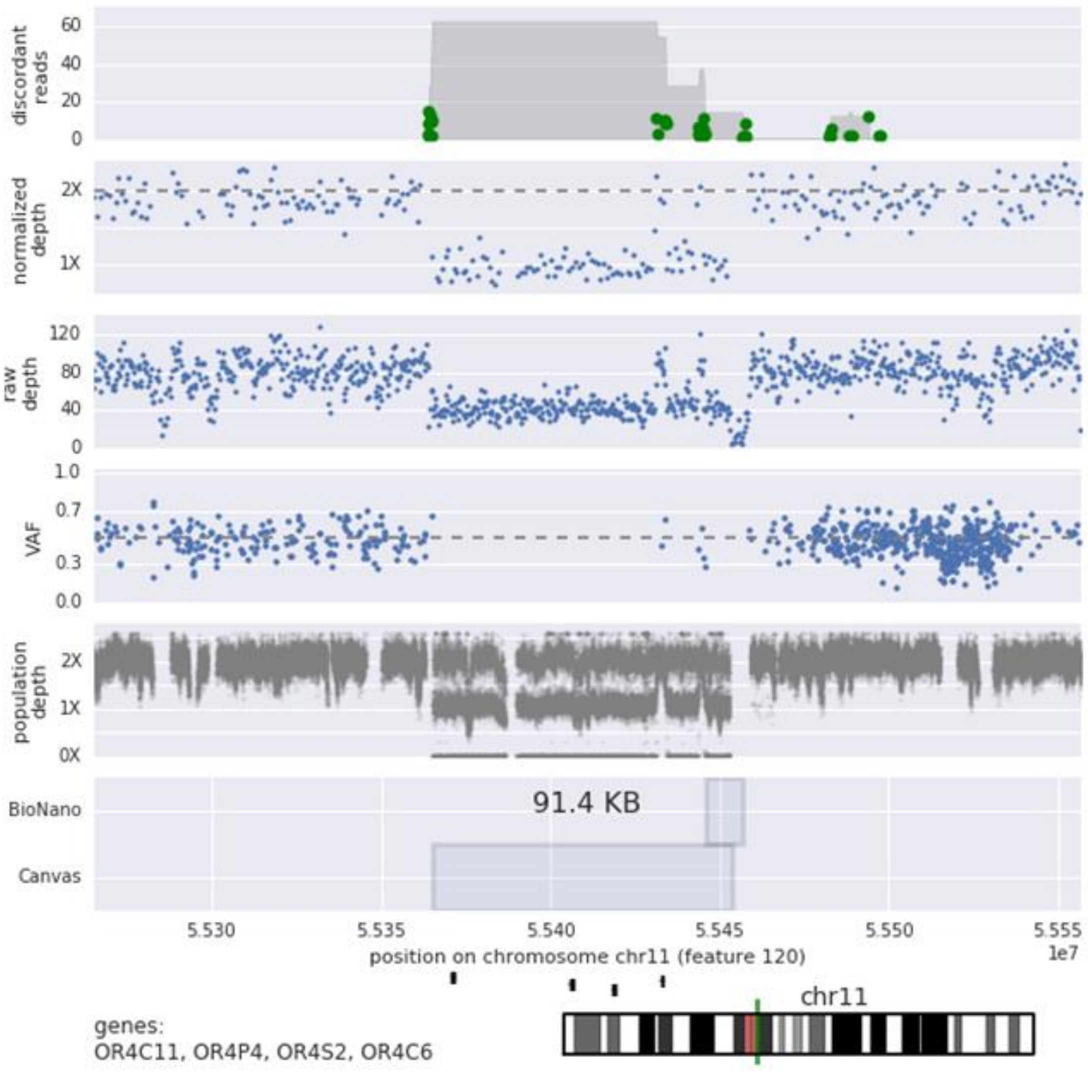

Supplemental Note Figure 3: This is a very common deletion supported by both population data, as well as discordant sequencing reads. The PacBio/BioNano call-set only partially called this deletion, but the data strongly support the GS depth based call. We suspect that this was missed by the alternative technologies due to the presence of more complex structural rearrangement in the region.

Manual Inspection of Coriell CNV Calls

We conducted an investigation of false negative (FN) calls to determine if any systematic issues could be identified. To search for error modes, FN calls were analyzed via manual inspection of microarray depth, sequencing depth, and discordant reads. We found nearly all of the discrepant calls occurred in low complexity regions not covered by microarray, or had ambiguous annotation on the Coriell website and/or copy number calling publication[3]. Although we cannot definitively conclude that certain calls from Coriell are erroneous, data from NGS and multiple genotyping arrays do not support a majority of these calls. To this effect, while the initial recall was calculated at 86% (31/36) events, this in-depth view of data leads us to speculate that the sensitivity is considerably higher.

Manual Inspection of Coriell CNV Calls

Prior to validation, a 75% reciprocal overlap threshold was set for calling of concordant calls. In **Table 1** we note 4 CNV calls with reciprocal overlaps in the range of 50-75%. A post-hoc analysis of this data generally support the boundaries of the Canvas CNV. The Coriell provided coordinates for all four CNVs are provided in Table S1.

NA02767: trisomy 21. The Coriell website records the CNV as extending across the centromere, whereas canvas calls the trisomy as the entirety of 21q, resulting in a 70% overlap. We note that we cannot call CNVs into the 21p due to low sequence complexity.

NA06804: HPRT1 duplication. The Coriell website reports a qualitative description of exon 2 and 3 duplication. The Canvas call is shifted from the truth-set coordinates by 3kb, but is well supported by both the read depth, as well as discordant read data. See **Supplemental Note Figure 4.**

NA20304: 15q GAIN. This CNV is a common GAIN of genetic material near the centromere of chromosome 15. Canvas split this into two separate calls with a large gap in the middle due to low complexity sequence as shown in **Supplemental Note Figure 5**.

ND01037: PARK2 exon 4 deletion. Inspection of sequencing depth data clearly supports the GS based coordinates for this homozygous deletion. See **Supplemental Note Figure 6**.

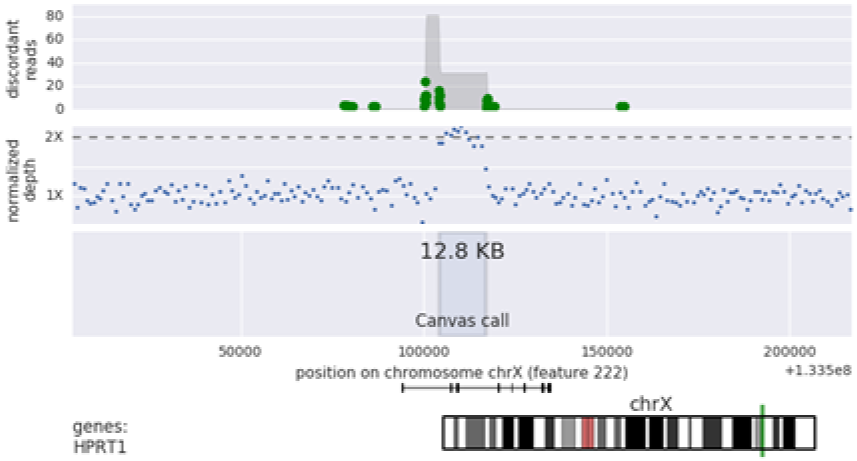

Supplemental Note Figure 4: Depth and discordant read locations in Coriell sample NA06804 near the HPRT1 locus.

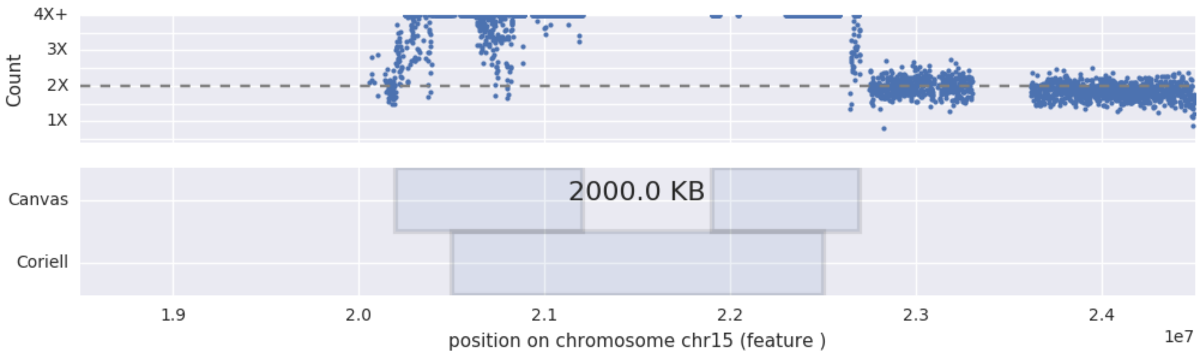

Supplemental Note Figure 5: Depth in Coriell sample NA20304 near the centromere on 15q.

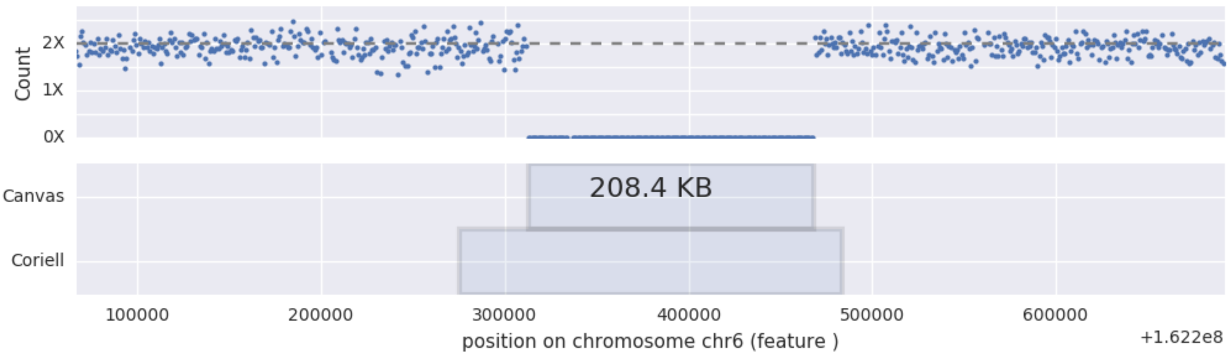

Supplemental Note Figure 6: Depth in Coriell sample ND01037 near the PARK2 exon 4 deletion.

Manual Inspection of Calls with Less Than 75% Reciprocal Overlap

For assessment of reference call recovery, we chose a one-sided overlap to assess the fraction of a given reference call recovered. This was due to many of the reference CNVs in Coriell being reported by exon- or probe-based measurements and/or compatibility issues between reference assemblies resulting in imprecise CNV boundaries. In addition, a bi-directional metric is complicated by the presence of reference call CNVs being represented by multiple CNVs from our calling pipeline. This is unavoidable for larger CNVs as benign variation often breaks up large CNVs into multiple calls: for example for the case of trisomy 21, there were multiple benign deletions causing a deviation from copy number 3 within much smaller regions.

During our validation, calls were manually curated to check for the type of edge cases where a large artefactual CNVs, may spuriously validate a reference call. To formally assess this, CNVs overlapping reference set calls were assessed for the fraction of the call overlapping the reference call. Nine calls with less 75% overlap were curated, and are represented below. It can be seen that these are clearly supported by the sequencing depth data and unlikely to be artifacts. See **Supplemental Note Figures 5 and 7-14.**

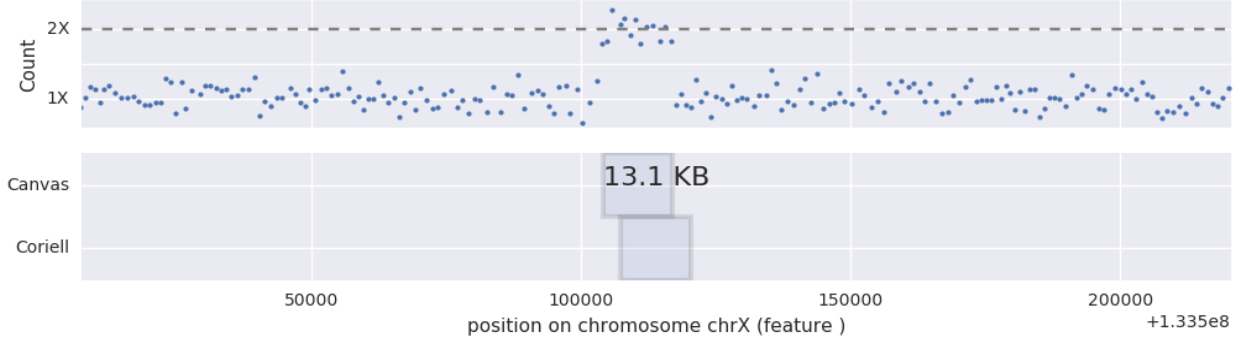

Supplemental Note Figure 7: Depth in Coriell sample NA06804.

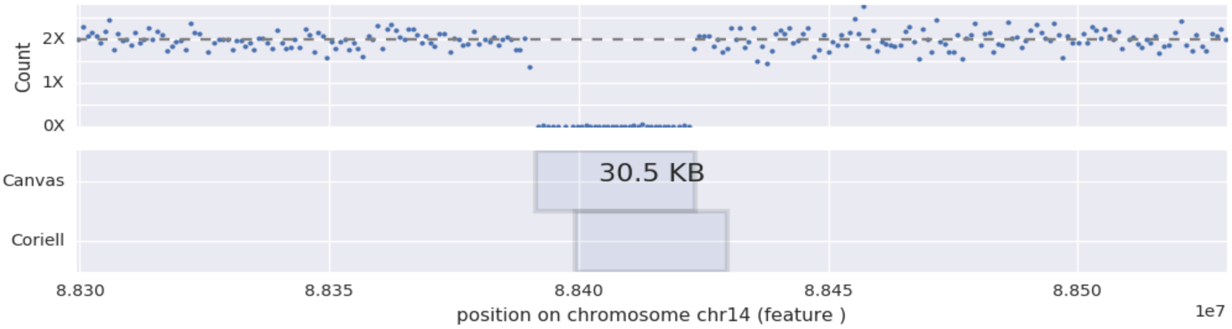

Supplemental Note Figure 8: Depth in Coriell sample NA04517.

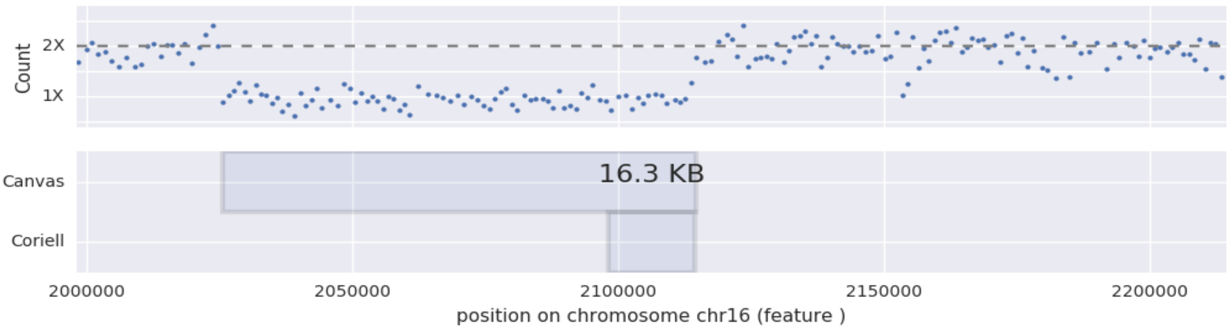

Supplemental Note Figure 9: Depth in Coriell sample NA04520.

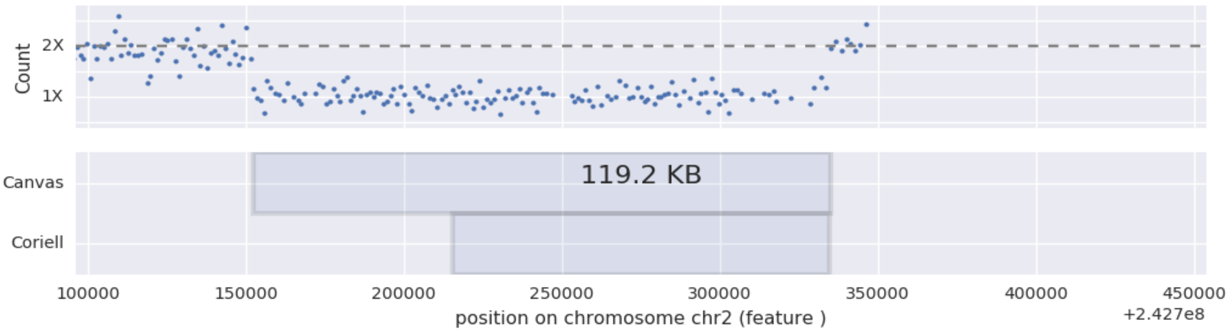

Supplemental Note Figure 10: Depth in Coriell sample NA13590.

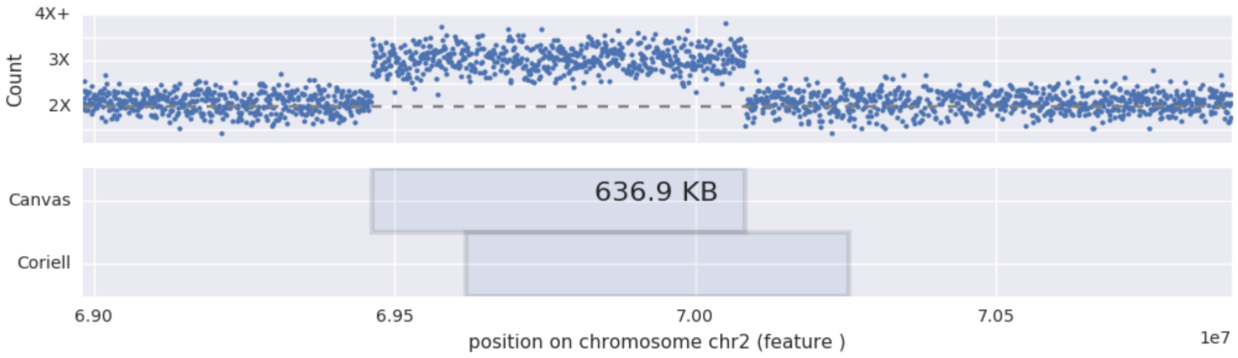

Supplemental Note Figure 11: Depth in Coriell sample NA16451.

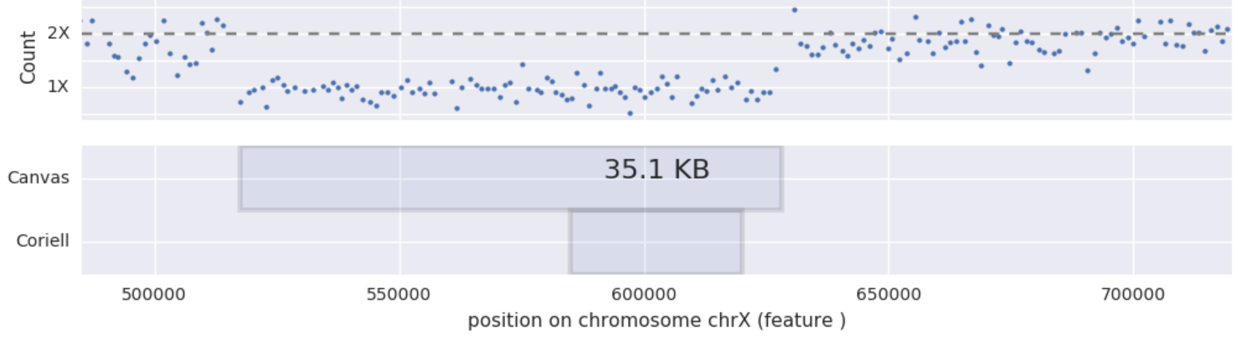

Supplemental Note Figure 12: Depth in Coriell sample NA20217.

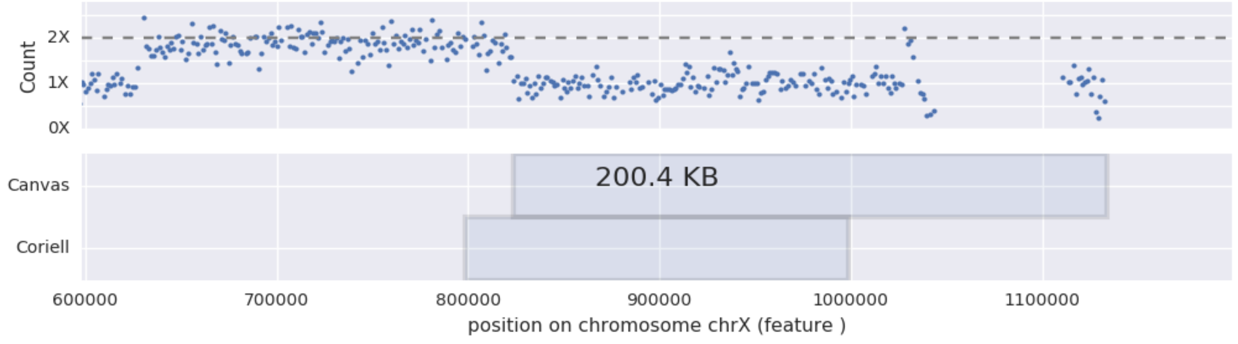

Supplemental Note Figure 13: Depth in Coriell sample NA20217.

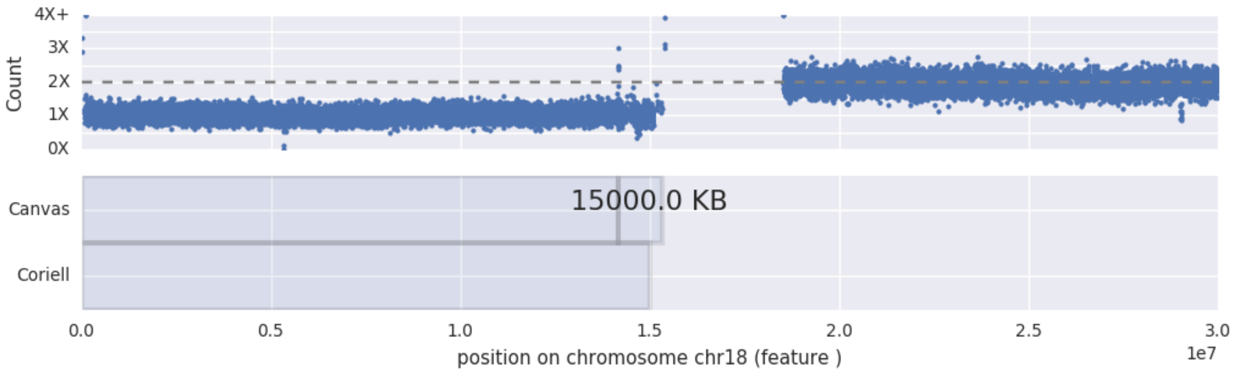

Supplemental Note Figure 14: Depth in Coriell sample NA21886. NA21886: This CNV is a loss of 18p and was split into two calls by our CNV Caller. The smaller call extends past the boundary of the reference call, but qualitatively it is clear that the correct call should extend to the centromere.

Manual Inspection of False Negative Coriell CNV Calls

Independent investigation of false negative (FN) calls was performed to determine if any systematic issues could be identified (**Supplemental Note Table 1**). To search for error modes, FN calls were analyzed via manual inspection of microarray depth, sequencing depth, and discordant reads.

Supplemental Note Table 1: Investigation of false negative Coriell CNV calls.

Coriell Call	Subject	CHROM	Size	Coriell CN	Array evidence	Manual inspection evidence	Suspected reason for FP
**6**	NA06804	chrX	13	0	No	No	Misinterpretation of primary data
**7**	NA09834	chr19	105	1	No	No	Poor mapping or array artifact
**30**	NA20304	chr15	418	1	No	No	Poor mapping or array artifact
**33**	NA21886	chr2	102	3	No	No	Bad annotation in Coriell
**37**	NA21886	chr4	112	1	No	No	Bad annotation in Coriell

One false negative from the Coriell call-set call occurred in the NA6804 sample on the first intron of the HPRT1 gene. Re-inspection of the literature supporting this event showed conflicting reports of this pathogenic rearrangement. Yang et al.[4,5] report a duplication of exons 2 and 3 of the gene alongside a deletion of exon 1. In contrast Monnat et al.[6] showed that the gain in exons 2/3 results from an insertion of the sequence into the first intron of HPRT1. While Canvas correctly identified the reported duplication, the read depth and paired read data seem to support the latter report of an insertion of this sequence into the first intron (**Supplemental Note Figure 4**). Based off of this evidence as well as Monnet et al., it is likely that the reported deletion is actually an artifact of the experimental methodology of Yang et al. as opposed to a true CNV.

The remaining four discrepancies indicate that the Coriell calls could be the results of artifacts in the experimental or bioinformatic analysis of these cell lines, or could be events originating at the cell line level that have diverged between different cell line specimens. Among the two discordant CNVs in NA21886, there is good coverage of GS sequencing data and array probes but not evidence of a CNV (**Supplemental Note Figure 15)**. In contrast, the CNVs on NA09834 and NA20304 are likely mapping or array artifacts. Take for example the 418kb deletion on chromosome 15 in NA20304 (**Supplemental Note Figure 16**). This CNV is reported in hg18 coordinates, and in our arrays it seems as though there are few probes within the region. Looking at the mappability (UCSC track 'Duke unique 35mers') between hg18 and hg19 in this region, it becomes clear that the updated reference has the sequence of this region represented multiple times as the 'uniqueness' drops from ~1 to ~.5 in most of the region (**Supplemental Note Figure 16)**. Thus we can conclude that in hg18, this is likely a copy-number 4 region in the reference and any copy-number variants would represent this collapsed representation. Taken together we hypothesize that this CNV could be an artifact of the Affymetrix array from which it was derived, but have insufficient evidence to definitively rule this call out as a false negative.

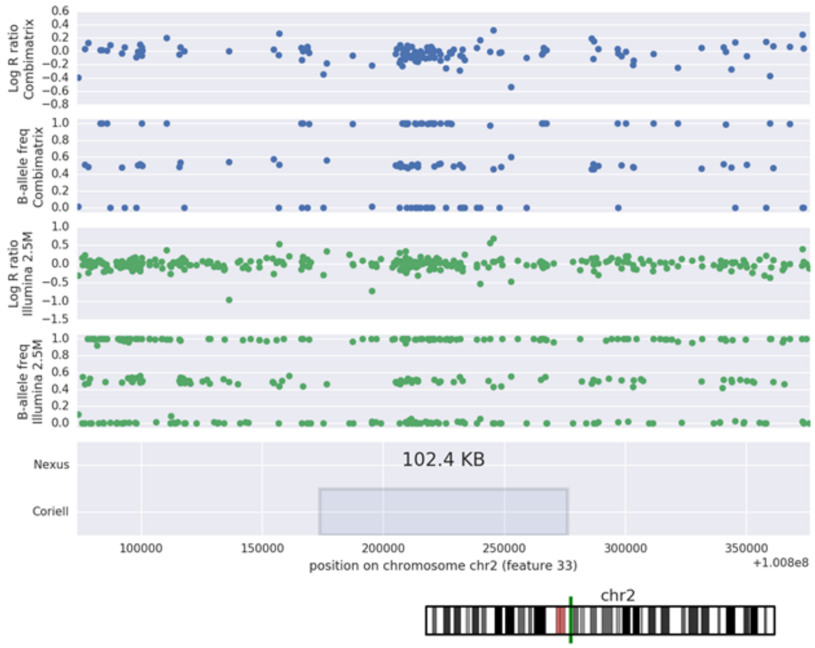

Supplemental Note Figure 15: Example of CNV annotated in the Coriell sample NA21886 that has little to no support from two commonly used clinical microarrays.

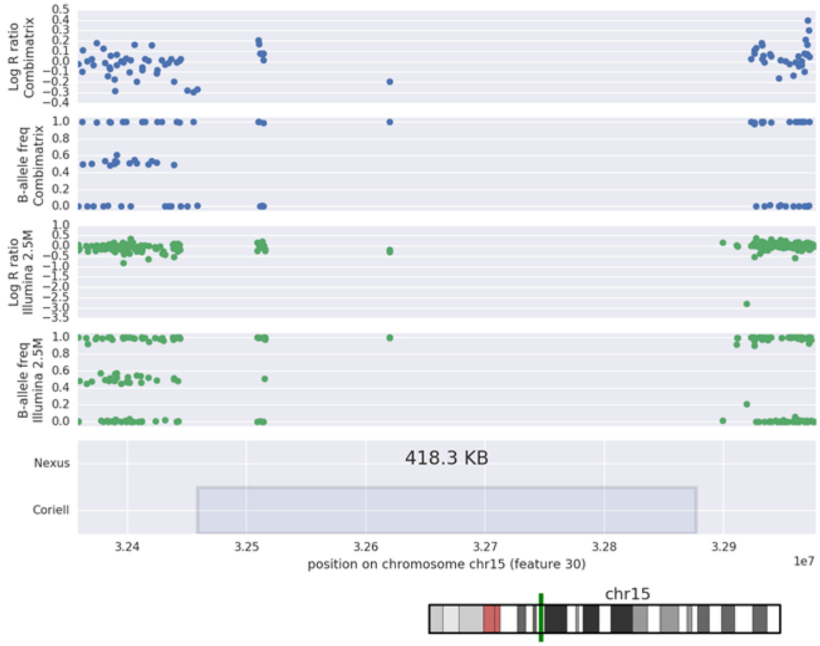

Supplemental Note Figure 16: Example of CNV annotated in the Coriell sample NA20304 that has little to no support from two commonly used clinical microarrays.

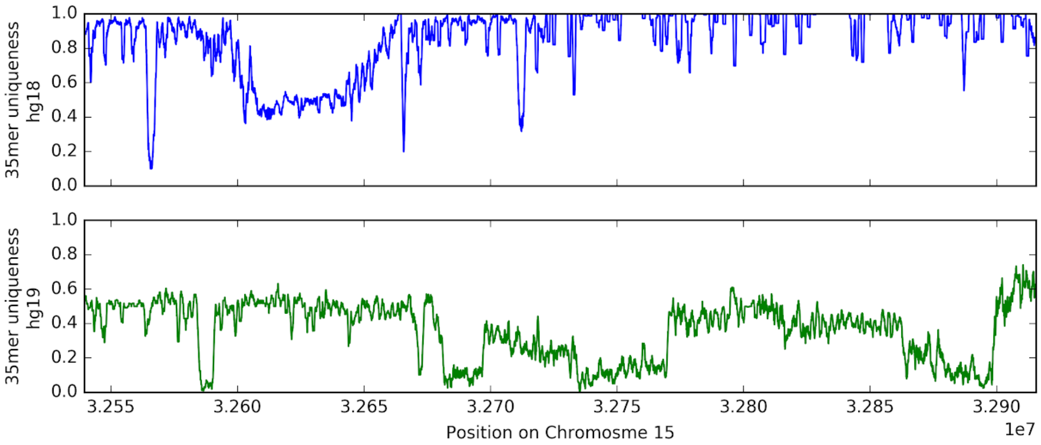

Supplemental Note Figure 17: Uniqueness of reference genome sequence across hg18 and hg19 reference assemblies. Plotted here is a 1000bp rolling window.

Microarray confirmation of CNVs

All sequenced clinical samples were run in parallel with Illumina Infinium Omni 2.5 genotyping chips. Array-based CNV analysis was conducted post-hoc on all samples with a positive CNV result that was not validated externally. Samples were processed in a single batch through GenomeStudio, and median centered across the cohort. All listed p-values are assessed via a permutation of the probe logR values for a given sample for all autosomes except for that on which the CNV resides. Note that the CNVs reported in subjects P6 and P7 were confirmed by an external clinical microarray lab, and the P16 was confirmed via karyotype.

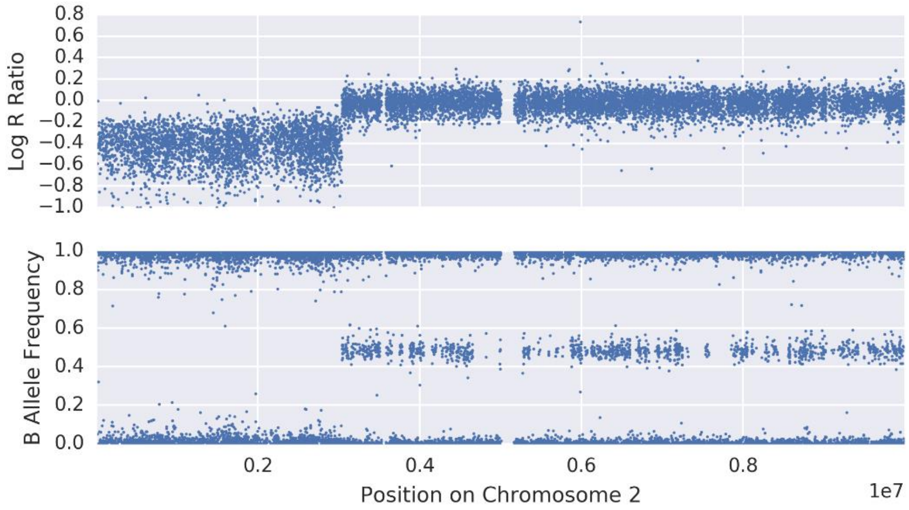

Supplemental Note Figure 18: Subject P7. chr2: 11314- 3033976. Empirical $P < 10^{-10}$.

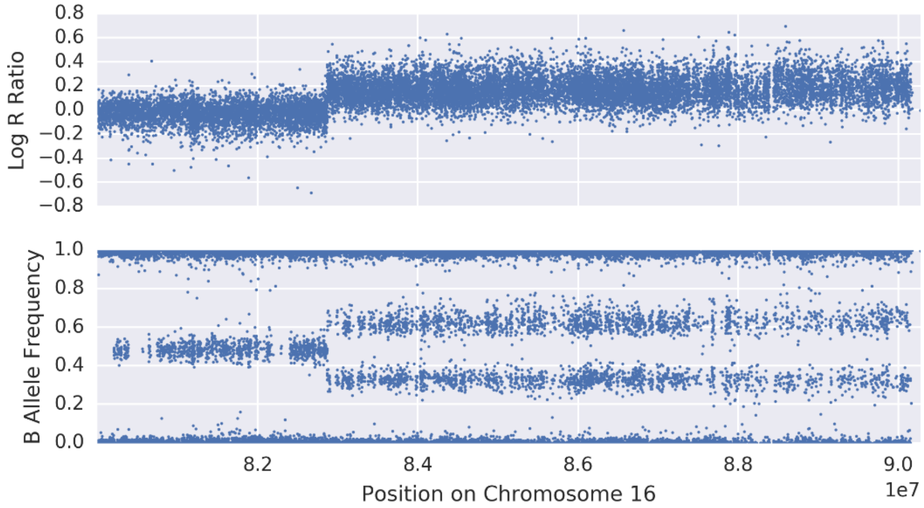

Supplemental Note Figure 19: Subject P7. chr16: 82865402- 90163542. Empirical $P < 10^{-10}$.

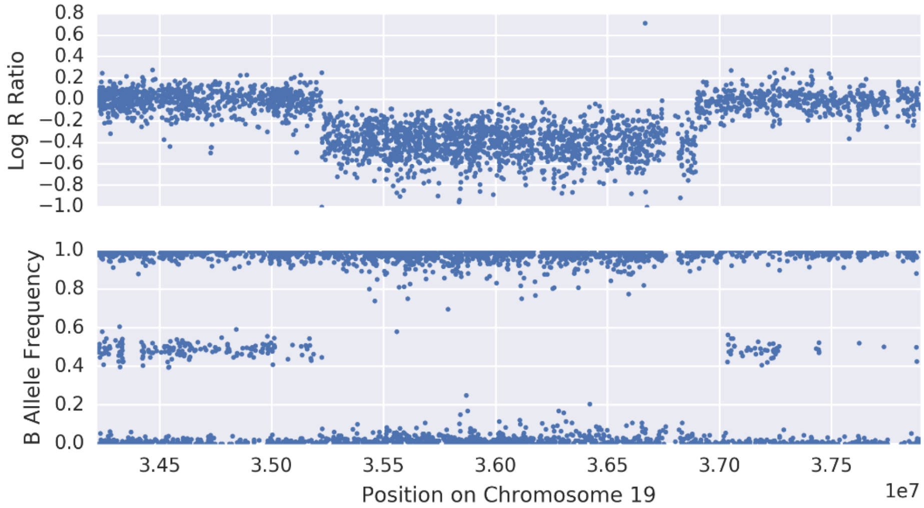

Supplemental Note Figure 20: Subject P8. chr19: 35223021- 36895699. Empirical $P < 10^{-10}$.

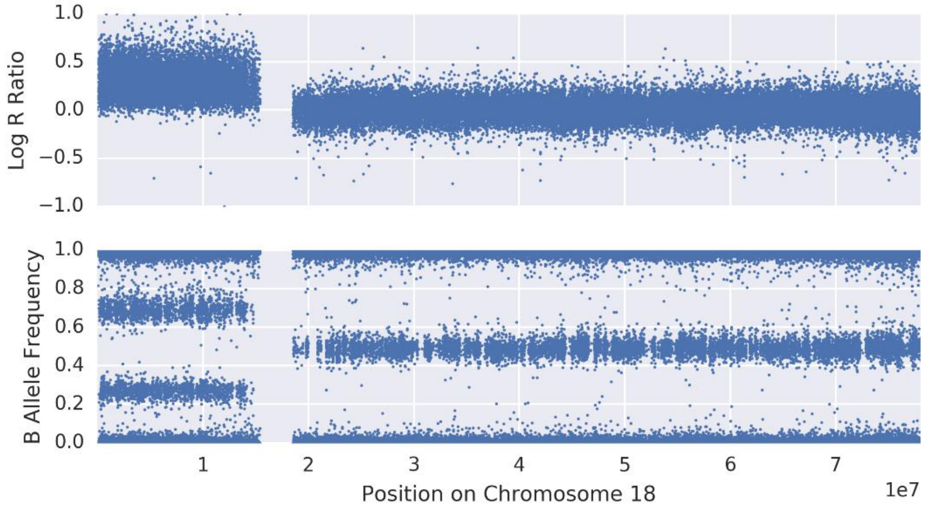

Supplemental Note Figure 21: Subject P9. chr18: 11494- 15404287. Empirical $P < 10^{-10}$.

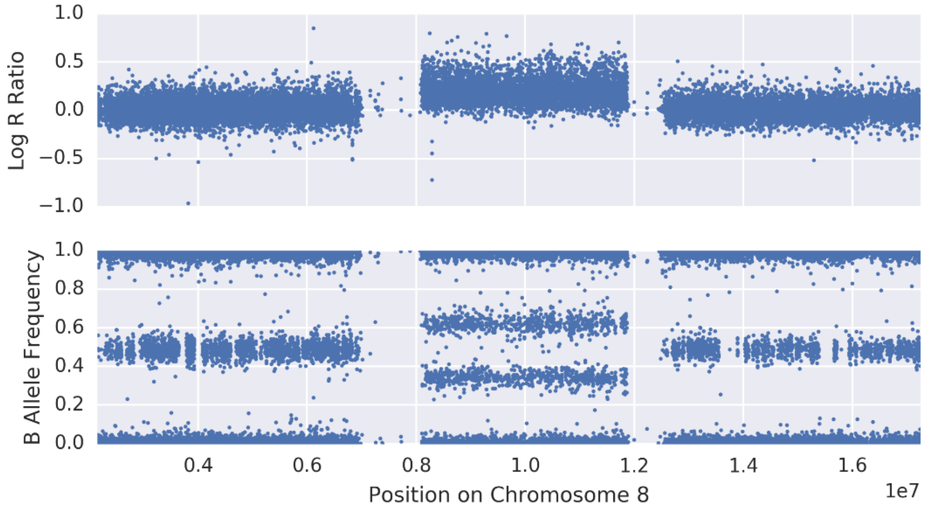

Supplemental Note Figure 22: Subject P10. chr8: 7153587- 12245784. Empirical $P < 10^{-10}$.

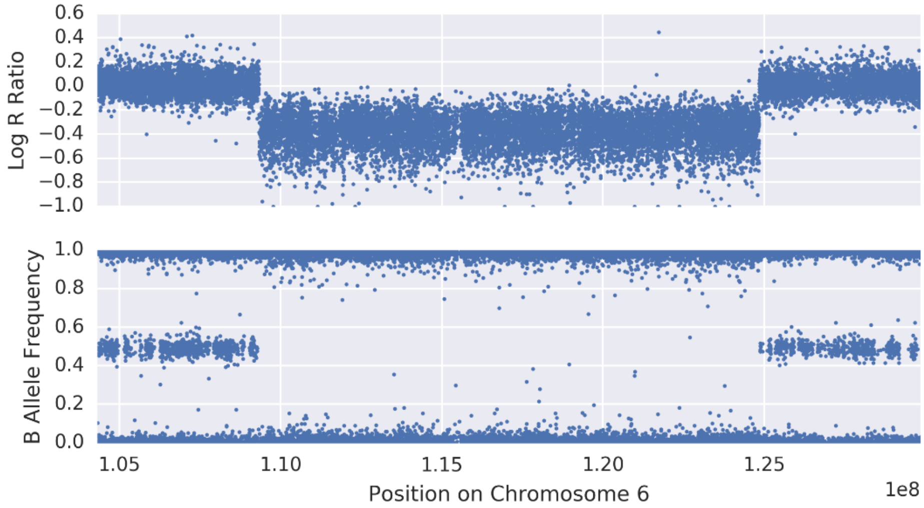

Supplemental Note Figure 23: Subject P11. chr6: 109324789- 124836619. Empirical $P < 10^{-10}$.

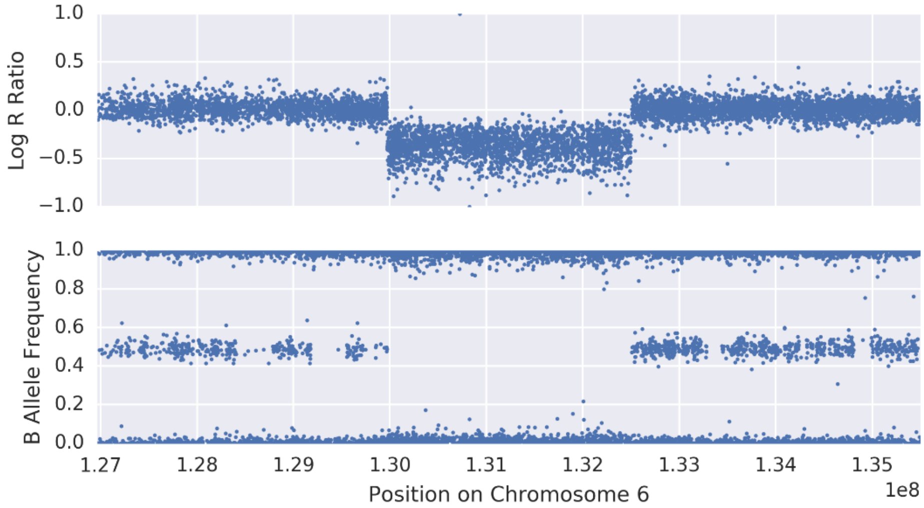

Supplemental Note Figure 24: Subject P11. chr6: 129969121- 132499298. Empirical $P < 10^{-10}$.

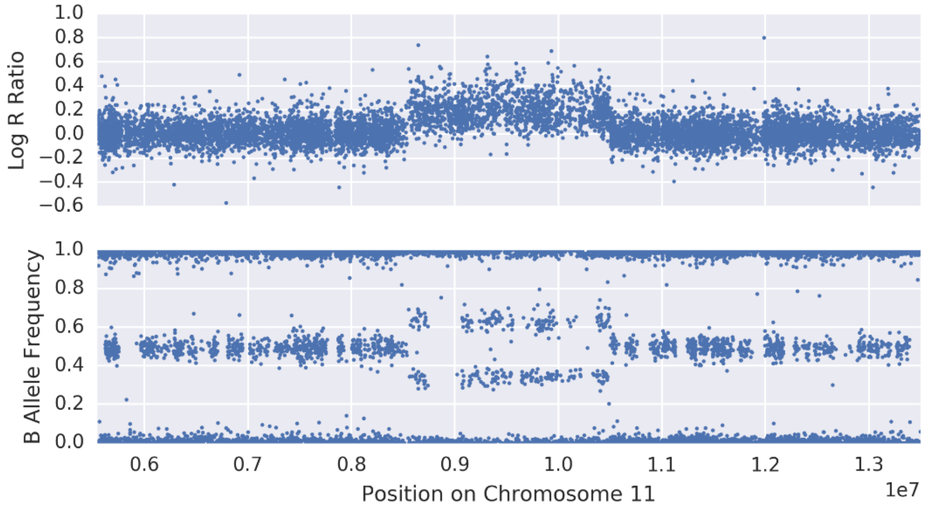

Supplemental Note Figure 25: Subject P11. chr11: 8548056- 10497905. Empirical $P < 10^{-10}$.

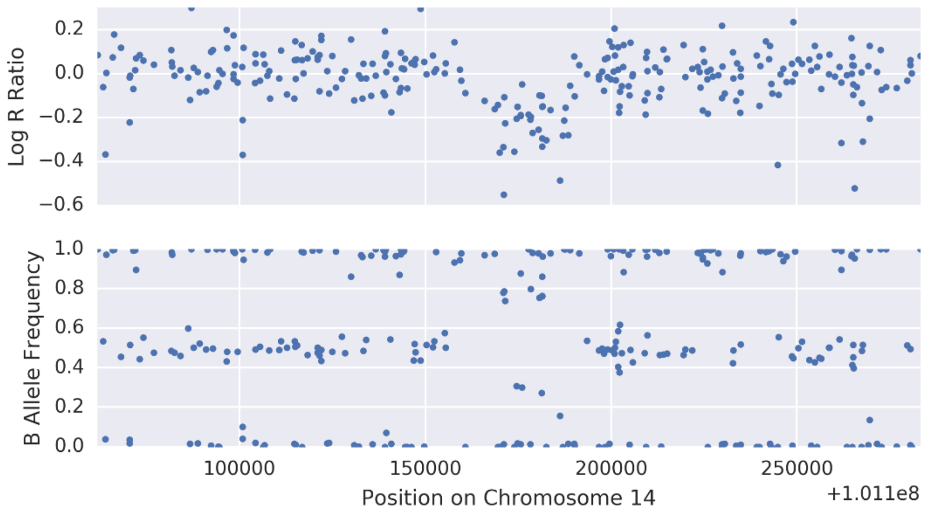

Supplemental Note Figure 26: Subject P12. chr14: 101261679- 101288013. Empirical $P < 0.0002$.

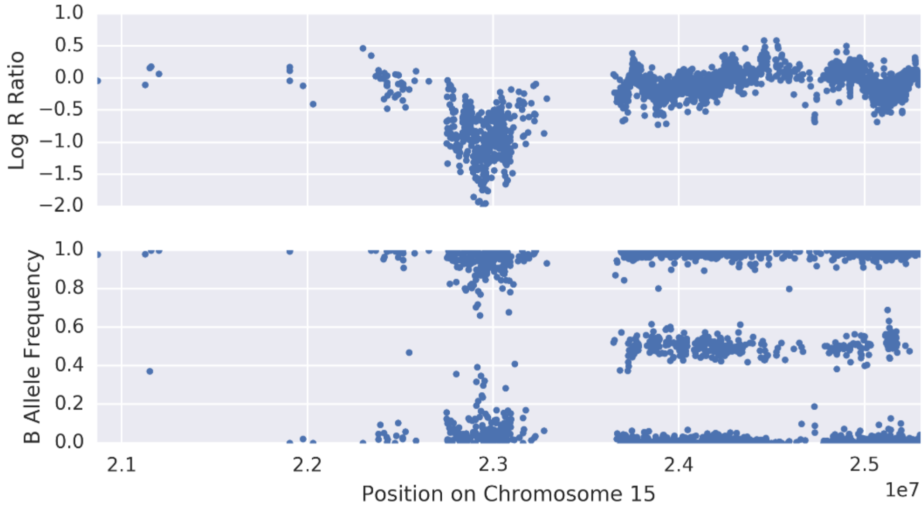

Supplemental Note Figure 27: Subject P13. chr15: 22696624- 23301066. Empirical $P < 0.01$. Note that this was a low quality array sample (LogRDev = 0.33), which was deemed suitable for sample tracking but would not normally be used in CNV analysis. We note that in addition to the significant depth change, we also see an absence of heterozygous variants which lends further support to the deletion call.

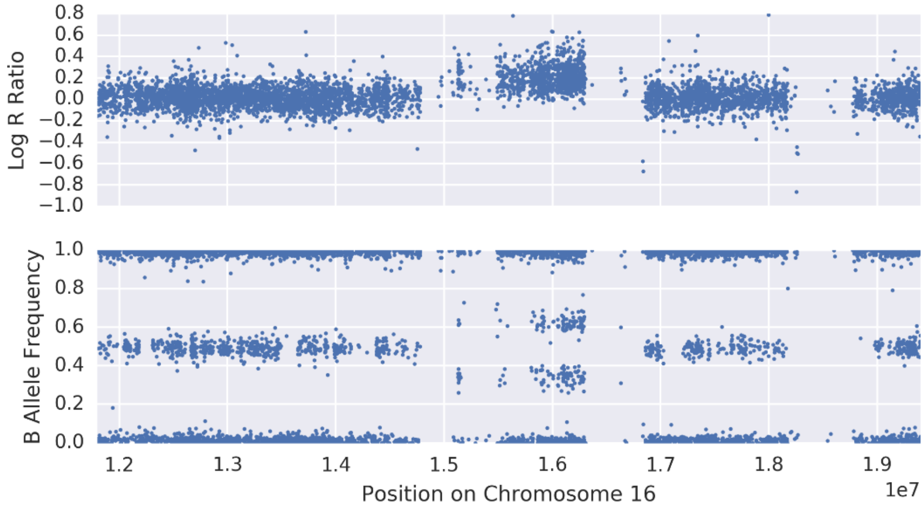

Supplemental Note Figure 28: Subject P14. chr16: 14800000- 16400000. Empirical $P < 10^{-10}$.

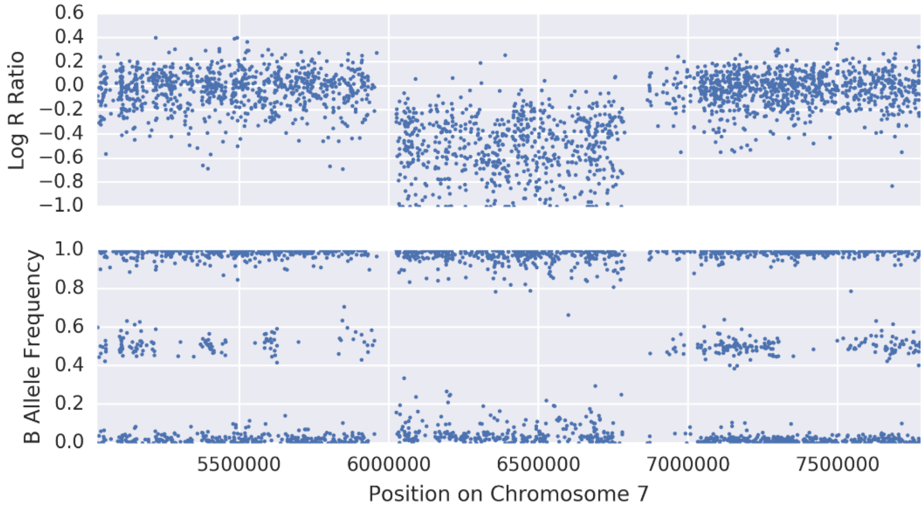

Supplemental Note Figure 29: Subject P15. Chr7: 6027017- 6776186. Empirical $P < 10^{-10}$.

de novo CNV phasing models

For de-novo CNVs we observe the inheritance patterns of small variants to decipher parental haplotype on which a CNV resides.

Deletion phasing

Here we simply compare inheritance of variants under the assumption of the deletion being on either the maternal or paternal alleles.

Supplemental Note Table 2: Model assuming deletion on paternal allele (all variants inherited from mother):

mother	father	CN-0	CN-1
0/0	0/1	1.0	0.0
	1/1	1.0	0.0
0/1	0/0	0.5	0.5
	0/1	0.5	0.5
	1/1	0.5	0.5
1/1	0/0	0.0	1.0
	0/1	0.0	1.0

Example deletion:
3MB deletion on the paternal allele. See Supplemental Note Figure 8 for illustration of model transition frequencies.

Model log-likelihoods:

```
father          -2472.071702
mother          -6295.203394
```

Prediction: *de novo* deletion on paternal allele.

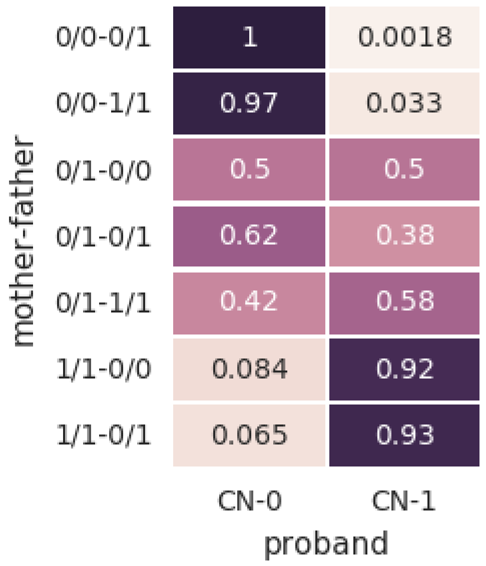

Supplemental Note Figure 8: Transition frequencies for example deletion.

Gain phasing

For gains, there are four possible scenarios. A gain may be of maternal or paternal origin, and be either simple or complex. By simple we refer to a duplication of a single allele, while a complex gain refers to the scenario where a proband can inherit material from both parents' copies of the DNA segment (an example of this is in an unbalanced translocation). Additionally, rather than having two copy states as in the case of deletions, gains have four possible variant copy states.

Supplemental Note Table 3: Model assuming a simple duplication of a maternal allele:

mother	father	CN-0	CN-1	CN-2	CN-3
0/0	0/1	0.50	0.50	0.00	0.00
	1/1	0.00	1.00	0.00	0.00
0/1	0/0	0.50	0.00	0.50	0.00
	0/1	0.25	0.25	0.25	0.25
	1/1	0.00	0.50	0.00	0.50
1/1	0/0	0.00	0.00	1.00	0.00
	0/1	0.00	0.00	0.50	0.50

Supplemental Note Table 4: Model assuming inheritance of **both maternal alleles** (along with one paternal allele):

mother	father	CN-0	CN-1	CN-2	CN-3
0/0	0/1	0.5	0.5	0.0	0.0
	1/1	0.0	1.0	0.0	0.0
0/1	0/0	0.0	1.0	0.0	0.0
	0/1	0.0	0.5	0.5	0.0
	1/1	0.0	0.0	1.0	0.0
1/1	0/0	0.0	0.0	1.0	0.0
	0/1	0.0	0.0	0.5	0.5

Example gain:

7MB deletion on the paternal allele. Allele fraction across genotypes clearly shows the dependence of copy number state on parental genotypes (**Supplemental Note Figure 9**).

Model likelihoods:

```
father-complex        -7948
father-dup            -26755
mother-complex        -19933
mother-dup            -24622
```

Prediction: The gain is resultant from inheritance of both paternal alleles, along with a single maternal allele.

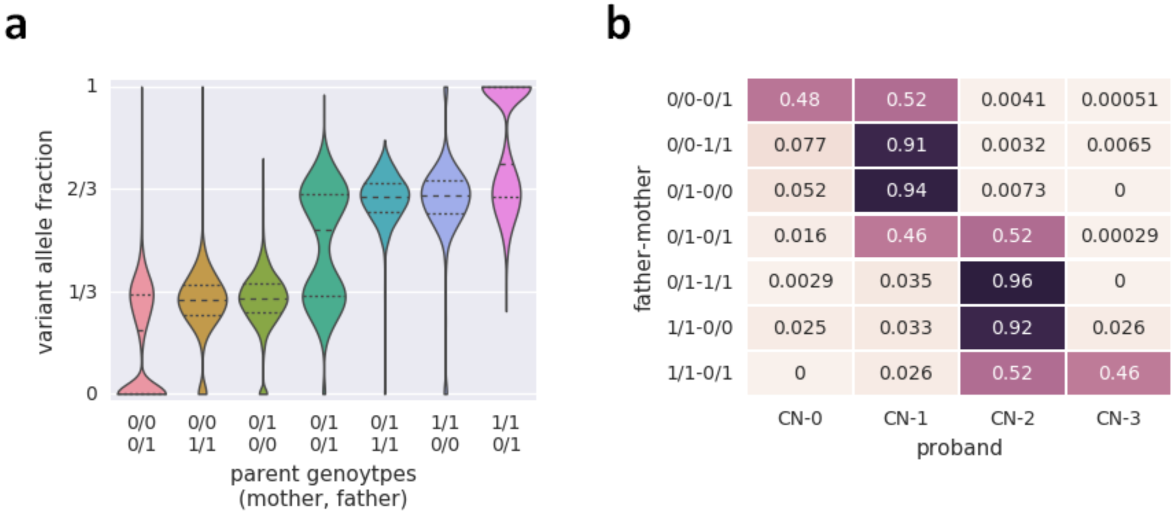

Supplemental Note Figure 9: Variant allele fraction across parental genotypes (**a**) and copy-number state transition frequencies (**b**) for example duplication.

Supplemental References

1. Sudmant PH, Rausch T, Gardner EJ, et al. An integrated map of structural variation in 2,504 human genomes. *Nature*. 2015;526(7571):75-81.

2. Spies N, Zook JM, Salit M, Sidow A. svviz: a read viewer for validating structural variants. *Bioinformatics*. 2015;31(24):3994-3996.

3. Tang Z, Berlin DS, Toji L, et al. A dynamic database of microarray-characterized cell lines with various cytogenetic and genomic backgrounds. *G3* . 2013;3(7):1143-1149.

4. Yang TP, Patel PI, Chinault AC, et al. Molecular evidence for new mutation at the hprt locus in Lesch-Nyhan patients. *Nature*. 1984;310(5976):412-414.

5. Yang TP, Stout JT, Konecki DS, Patel PI, Alford RL, Caskey CT. Spontaneous reversion of novel Lesch-Nyhan mutation by HPRT gene rearrangement. *Somat Cell Mol Genet*. 1988;14(3):293-303.

6. Monnat RJ Jr, Chiaverotti TA, Hackmann AF, Maresh GA. Molecular structure and genetic stability of human hypoxanthine phosphoribosyltransferase (HPRT) gene duplications. *Genomics*. 1992;13(3):788-796.
